# Tunable optical and semiconducting properties of eco-friendly-prepared reduced graphene oxide

**DOI:** 10.3389/fchem.2023.1267199

**Published:** 2023-08-31

**Authors:** Talia Tene, Yuliana Jiménez-Gaona, Diana Katherine Campoverde-Santos, Yesenia Cevallos, Matteo La Pietra, Cristian Vacacela Gomez, Andrea Scarcello, Salvatore Straface, Lorenzo S. Caputi, Stefano Bellucci

**Affiliations:** ^1^ Department of Chemistry, Universidad Técnica Particular de Loja, Loja, Ecuador; ^2^ Facultad de Ciencias Pecuarias, Escuela Superior Politécnica de Chimborazo (ESPOCH), Riobamba, Ecuador; ^3^ College of Engineering, Universidad Nacional de Chimborazo, Riobamba, Ecuador; ^4^ Universidad San Francisco de Quito, Quito, Ecuador; ^5^ INFN-Laboratori Nazionali di Frascati, Frascati, Italy; ^6^ Department of Information Engineering, Polytechnic University of Marche, Ancona, Italy; ^7^ UNICARIBE Research Center, University of Calabria, Cosenza, Italy; ^8^ Surface Nanoscience Group, Department of Physics, University of Calabria, Cosenza, Italy; ^9^ Department of Environmental Engineering (DIAm), University of Calabria, Cosenza, Italy

**Keywords:** reduced graphene oxide, ascorbic acid, optical bandgap, absorption coefficient, I-V curves

## Abstract

Wide bandgap oxidized graphenes have garnered particular interest among the materials explored for these applications because of their exceptional semiconducting and optical properties. This study aims to investigate the tunability of the related properties in reduced graphene oxide (rGO) for potential use in energy conversion, storage, and optoelectronic devices. To accomplish this, we scrutinized crucial parameters of the synthesis process such as reduction time and temperature. Our findings demonstrate that controlling these parameters makes it possible to customize the optical bandgap of reduced graphene oxide within a range of roughly 2.2 eV–1.6 eV. Additionally, we observed that reduced graphene oxide has strong and superior absorption in the visible region, which is attributable to the existence of OFGs and defects. Notably, our results indicate that the absorption coefficients of reduced graphene oxide are up to almost three times higher (7426 ml mg^−1^ m^−1^) than those observed in dispersions of exfoliated graphene and graphene oxide (GO). To complement our findings, we employed several spectroscopic and morphological characterizations, including scanning electron microscopy (SEM), energy-dispersive X-ray spectroscopy (EDS), X-ray diffraction (XRD), and electrical measurements. The implications of our results are significant for the development and design of future semiconductors for energy conversion and optoelectronic applications.

## 1 Introduction

Graphene, a two-dimensional (2D) material, consists of a single layer of carbon atoms arranged in a hexagonal honeycomb-like lattice (Yee and Ghayesh, 2023). It serves as a foundational building block for other carbon-based materials, including graphite and carbon nanotubes. Graphene exhibits exceptional properties, such as high thermal and electrical conductivity, mechanical strength, and a large surface area (Liu et al., 2023). However, there are challenges associated with pristine graphene, as it is insoluble in liquid media (Wu W. et al., 2023), limiting its ease of handling and integration into advanced devices. Moreover, being a zero-bandgap material, graphene behaves like a semimetal rather than a semiconductor and lacks functional groups that offer advantages for bio-applications (Demirel and Durmaz, 2023).

In this way, graphene derivatives offer promising solutions to address the limitations of pristine graphene (Wu J. et al., 2023). Among these derivatives, graphene oxide (GO) stands out, as it is obtained by introducing oxygen functional groups (OFGs) to the graphene lattice, including hydroxyl, epoxy, and carboxyl groups (Ferrari et al., 2023). This process not only imparts hydrophilicity to GO but also facilitates easier handling. Moreover, the introduction of OFGs in GO results in the creation of a finite and wide bandgap, transforming it into a semiconductor or insulating material (Michels Brito et al., 2023). On the other hand, reduced graphene oxide (rGO) is produced by reducing GO, which leads to the removal of OFGs and restoration of some of the electrical and mechanical properties found in pristine graphene (Chen et al., 2023). Both GO and rGO exhibit distinctive properties that render them valuable for a wide range of applications, including energy storage, biosensors, and smart coatings (Joshi et al., 2023; Manikandan and Lee, 2023).

The physical and chemical properties of GO and rGO can be influenced by the oxidation process and the choice of reductant agent (Wu J. et al., 2023). Different oxidation methods, including the Hummers method (Shahriary and Athawale, 2014), improved Hummers method (Marcano et al., 2010), and eco-friendly improved method (Chen et al., 2013), have been employed, while common reductant agents consist of hydrazine (Park et al., 2011), ascorbic acid (De Silva et al., 2018), and citric acid (Tene et al., 2022a). Despite significant research on synthesis processes and reducing agents, certain crucial parameters such as temperature and contact time with the reducing agent remain insufficiently explored. Recent efforts have been directed toward identifying key factors that allow for precise control of oxygen functionalization, which includes investigating aspects such as drying and reduction time (Gupta et al., 2017). This research is of great importance since it holds the potential to enable the development, mainly, of rGO with bandgaps comparable to silicon (around 1 eV). Such bandgaps would make GO and rGO well-suited for utilization in next-generation technologies (Gong et al., 2022).

Various methods, including chemical treatments and thermal annealing, have been proposed to modify the properties of GO (Wu J. et al., 2023). One particularly promising approach, introduced by Kumar et al. (Kumar et al., 2014), involves a mild thermal annealing procedure that transforms mixed sp^2^-sp^3^ hybridized GO phases into distinct oxidized and graphitic phases. This transformation leads to significant improvements and a strong increase in visible absorption characteristics. Notably, this method does not require any chemical treatments and can be carried out at temperatures ranging from 50°C to 80°C. However, while the study (Kumar et al., 2014) demonstrates the effectiveness of their thermal annealing method, it does not present specific results regarding the optical bandgap, electronic transitions, or absorption coefficient of the obtained materials. Additionally, the study does not explore the effects of this method on rGO.

In this context, in our previous study, we concentrated on addressing this lack of information specifically for GO (Tene et al., 2023a). However, additional research is essential to fully understand the potential of a similar approach for manipulating the semiconducting and optical properties of rGO.

Here, we investigated how temperature treatment and contact time with the reducing agent influence the semiconducting and optical properties of different rGO samples. To conduct our analysis, we utilized UV-visible spectroscopy to obtain absorbance spectra and employed the Tauc analysis to estimate the optical bandgap. Moreover, we employed spectroscopic and morphological characterizations to gain insights into the elemental composition, crystallinity, and surface structure. In particular, our research revealed that reducing GO using ascorbic acid (AA) at 80°C for 120 h can modify the optical bandgap of rGO from approximately 2.2 eV to around 1.6 eV. Furthermore, we provided a comprehensive and statistical discussion of the estimated values for the optical absorption coefficient, electronic transitions, and their relationship to the electrical conductivity of the obtained material.

## 2 Materials and methods

Here, we provide a brief description of the synthesis of GO and rGO, which is depicted in Schematic S1. Our prior works and associated applications (Tene et al., 2020; Tene et al., 2022b; Tene et al., 2023a; Tene et al., 2023b) contain more comprehensive information regarding the full synthesis process. Furthermore, for a meaningful comparison with the present work involving other environmentally friendly reducing agents, please refer to Ref. (Tene et al., 2023b).

### 2.1 Materials

In the following, we provide details of all the chemicals used as received without any additional purification during the oxidation and reduction process (see Table 1).

**TABLE 1:** Description of the chemical used for the synthesis of GO and rGO.

Chemical	Characteristics	Supplier
Graphite powder	<150 μ m, 99.9%	Sigma Aldrich
Sulfuric acid (H_2_SO_4_)	ACS reagent, 95.0%–98.0%	Sigma Aldrich
Potassium permanganate (KMnO_4_)	ACS reagent, 99.0%	Sigma Aldrich
Hydrochloric acid (HCl)	ACS reagent, 37%	Sigma Aldrich
Hydrogen peroxide (H_2_O_2_)	30%	Merk
Ascorbic acid	C_6_H_8_O_7,_ ≥99.5%	Sigma Aldrich

### 2.2 Synthesis of GO and rGO

The synthesis process involved adding 3.0 g of graphite powder to 70 mL of H_2_SO_4_ and cooling the mixture in an ice bath. Subsequently, 9 g of KMnO_4_ was added at a temperature below 20°C, and after 30 min, the mixture was stirred for an additional 30 min at 50°C. Distilled water (150 mL) was gradually added to the solution while maintaining the temperature below 90°C. Following this, an additional 500 mL of distilled water and 15 mL of H_2_O_2_ were added. The resulting mixture was divided into centrifuge tubes and washed with a 1:10 solution of HCl and distilled water through several centrifugations. The precipitate obtained was dried in an oven at 45°C for 48 h to obtain graphite oxide powder.

Then, 100 mg of the resulting powder was sonicated in 500 mL of distilled water to create a homogeneous GO suspension. After centrifugation, the suspension was dried at 80°C, and drying times were varied from 0 to 120 h.

In the reduction step, using the GO sample dried at 80°C for 24 h (see Ref. (Tene et al., 2023a). and discussions below), a 250 mL GO aqueous solution was prepared, and 500 mg of AA was added with stirring. Reduction times ranging from 1 to 120 h were tested at temperatures of 50°C and 80°C. To remove excess AA, the rGO precipitates were washed with distilled water by centrifugation. Finally, the precipitate was dried at 80°C for 24 h to obtain rGO powder. After each reduction period, the samples were sonicated to re-disperse GO or rGO before measuring their absorbance spectra.

### 2.3 Characterization

The absorption spectra of GO and rGO were recorded using a Jenway 6850 spectrophotometer. The wavelength range was set from 190 to 1000 nm with a resolution of 0.1 nm, and the optical absorption coefficient was calculated at λ = 660 nm. Quartz cuvettes with a 10 mm optical path and a volume of 3.5 mL were used. The spectra were normalized to the maximum of the prominent peak. Conventional Lorentz functions were applied for curve fitting, and a 7-point moving average was used to smooth the absorbance spectra.

To analyze the surface morphology of the samples, scanning electron microscopy (SEM) was employed with an accelerating voltage of 20 kV, and an energy-dispersive X-ray spectrometer (EDS) made by JEOL was used. X-ray diffraction (XRD) measurements were carried out using an X-ray diffractometer (PANalytical Pro X-ray) in the diffraction angle (2θ) range of 5°–90° with Cu Kα irradiation under an acceleration voltage of 60 kV and a current of 55 mA.

For electrical characterization, a KEI2450 instrument was used within a voltage window of 0–10 V.

## 3 Results and discussions

To further emphasize our eco-friendly approach, previous studies (Begum et al., 2017; Tene et al., 2022b; Tene et al., 2022c) have shown that GO can be transformed into rGO using green reducing agents such as citric acid (CA) (Tene et al., 2023b). In our current study, we specifically focus on GO reduced using AA. This choice represents a safer and more environmentally friendly alternative compared to using hydrazine, which poses potential health risks. Furthermore, we highlight that the parameters investigated in this study are the reduction time (from 1 to 120 h) and temperature (80°C and 50°C). These aspects are crucial for comprehending their influence on the optical and electrical properties of rGO.

### 3.1 The case of GO

Our work first concentrated on describing the key findings about the characteristics of GO under various drying times; for a thorough investigation, see Ref. (Tene et al., 2023a).

The absorption spectra of GO with drying times of up to 120 h are shown in Supplementary Figure S1. Two different absorption peaks can be detected, one at about 230 nm and the other at 300 nm (a shoulder peak). These peaks correspond to the $\pi -\pi^{*}$ transition of C=C in carbon systems, and a broad $n-\pi^{*}$ transition of C=O bonds, respectively (Schematic S1). All spectra are featureless in the visible region (see Supplementary Figure S2, spectrum-color bar from 400 to 700 nm).

A noteworthy observation is the redshift of the principal absorption peak, which was not previously reported and noted in (Kumar et al., 2014), from 229.6 nm at 0 h to 243.8 nm at 120 h (Supplementary Table S1). This result is intriguing because physisorbed water molecules on the GO structure are predicted to evaporate during drying (Tene et al., 2023a). The observed redshift of $\pi -\pi^{*}$ transitions may be explained by the fact that such evaporation would lead to a drop in the quantity of OFGs and a partial reduction of the material. However, considering the significant redshift, it is important to explore other potential factors or impurities that might contribute to this spectral change. For instance:

• Aggregation and stacking: Extended drying could lead to increased aggregation and stacking of GO sheets. This can alter the electronic interactions between the sheets, affecting the energy levels and transitions responsible for the absorbance peak.
• Reorganization of OFGs: Over longer drying times, the rearrangement of OFGs on the GO surface might occur. This could affect the energy levels of the material, leading to changes in its absorbance spectrum.
• Chemical reactions: Prolonged exposure to 80 °C might induce chemical reactions within the GO material. These reactions could involve the rearrangement, reduction, or further oxidation of functional groups, leading to shifts in the absorbance peak.
• Loss of volatile impurities: Besides physisorbed water molecules, there could be other volatile impurities or solvents present in the sample. Extended drying might lead to their removal, affecting the local environment of the GO and consequently its absorbance spectrum.
• Surface defects and rearrangement: The drying process might cause defects on the GO surface to rearrange or interact differently. These surface defects play a significant role in the electronic structure of the material and could contribute to changes in the absorbance peak.
• Strain effects: Extended drying might lead to mechanical strain within the GO sheets. This strain can impact the electronic band structure and result in shifts in the absorbance spectrum.

A widespread technique for determining the optical bandgap of GO is the Tauc method (Gul et al., 2023; Khan et al., 2023). This technique makes use of the Tauc analysis and the absorption spectrum of the material. In light of this, the linear component between 4 eV and 5 eV for each drying period was fitted to determine the optical bandgap of GO samples (Supplementary Figure S3). Supplementary Table S2 contains the numerical values for the estimated optical bandgap. The bandgap values decrease from 4.1 eV at 0 h to 2.8 eV at 120 h, showing a decreasing exponential trend as drying time rises. Between 0 and 24 h, drying time had the biggest effect, leading to a notable drop of 0.91 eV.

To calculate the absorption coefficient of GO, the Lambert-Beer law ( $A=\alpha_{660}∙c∙l$ ) is applied. This approach is usually used for exfoliated graphene dispersions in water or alcohol (Hernandez et al., 2008; Nicolosi et al., 2013). Keeping this in mind, several GO dispersions are made with concentrations ranging from 0.01 to 0.05 mg ml^−1^. The absorption coefficient of GO at different drying times was determined to be 3932.2 ml mg^−1^ m^−1^ at 0 h, 4586.7 ml mg^−1^ m^−1^ at 48 h, and 5507.2 ml mg^−1^ m^−1^ at 120 h (Supplementary Table S3). As an important outcome, the absorption coefficient of exfoliated graphene dispersions, which is typically around 2460 ml mg^−1^ m^−1^ (Hernandez et al., 2008), is much lower than the values found in GO.

Owing to the presence of OFGs, GO has a higher absorption coefficient than exfoliated graphene. Indeed, these functional groups introduce sp^3^-like defects into the carbon lattice, which can interact with photons at a larger range of energies. The OFGs on the surface of GO can also cause dipole moments and charge transfer, which improves electromagnetic radiation absorption even more. Because large absorption coefficients are crucial in applications such as photovoltaics and photocatalysis, GO appears as an effective material.

### 3.2 The case of rGO at 80 ℃

We now proceeded to examine the optical properties of rGO. For the reduction process, we focused on GO samples dried at 80 ℃ for 24 h, which showed the most efficient result from the drying process (Tene et al., 2023a). This GO sample resulted in a bandgap of 3.2 eV (Supplementary Table S2) and a $\pi -\pi^{*}$ transition at 233.3 nm (Supplementary Table S1).

We emphasize that the drying time significantly influences the electrical and optical properties of the resulting rGO, holding a pivotal role. For instance, consider the scenario of utilizing GO subjected to no drying (0 h), resulting in a material enriched with OFGs. In contrast, prolonging the drying time to 120 h yields a partially reduced material (Tene et al., 2023a). To mitigate this inherent variability and ensure the utmost objectivity in our current study, we have deliberately selected a GO sample dried at 80°C for 24 h. Non-etheless, for future investigations, it remains intriguing to commence with partially reduced samples, investigating the potential for achieving more extensive reduction beyond the outcomes detailed in this study.

Then, the absorption spectra of rGO reduced at 80°C using AA are shown in Supplementary Figure S5 for different reduction times ranging from 1 h to 120 h. In the visible region, all of the spectra are featureless, proving that no new species were added during the reduction process (Supplementary Figure S6). It is also interesting to notice that only the $\pi -\pi^{*}$ transition is detected, but the $n-\pi^{*}$ transition is not found in any of the spectra.

To comprehend these types of transitions, it is essential to revisit the principles of UV-Vis theory (Schematic S2). The $\pi -\pi^{*}$ transition is an electronic process where an electron in a π orbital is excited to a higher energy $\pi^{*}$ orbital. In rGO, this transition appears as a broad peak because of the delocalization of π -electrons in the sp^2^ hybridized carbon atoms forming the graphene lattice. In contrast, the $n-\pi^{*}$ transition occurs when an electron in a non-bonding ( n ) orbital is excited to a higher energy $\pi^{*}$ orbital. The absence of the $n-\pi^{*}$ transition in rGO is due to the removal of certain functional groups (e.g., C = O) during the reduction process, dropping the possibility of $n-\pi^{*}$ transitions.

**FIGURE 1:**
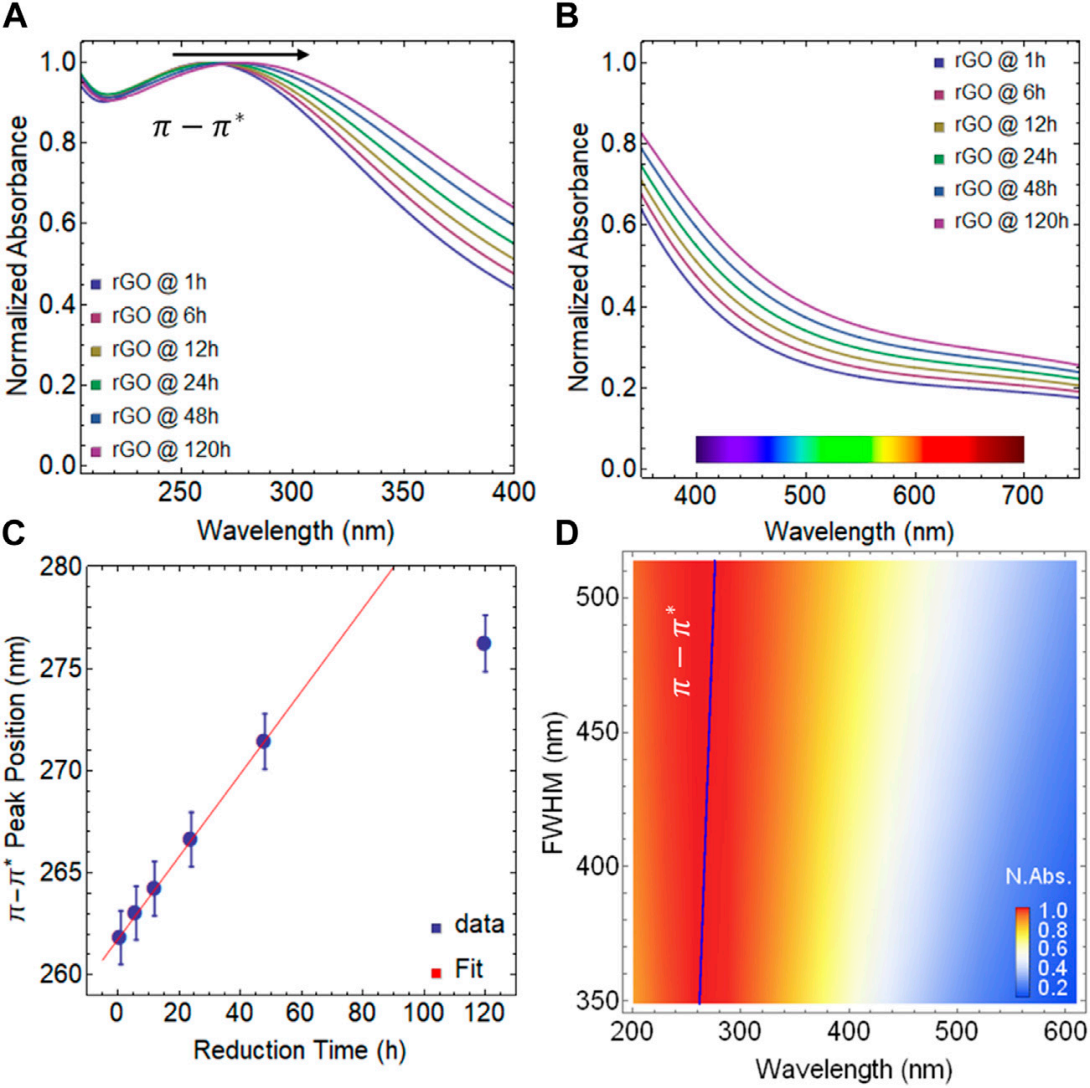
Absorbance spectra of rGO at 80 ℃ : **(A)** from 200–400 nm and **(B)** from 350–750 nm. **(C)** Position of $\pi -\pi^{*}$ transition as a function of reduction time. **(D)** Color plot: Normalized absorbance as a function of the FWHM and wavelength.

Hence, in Figure 1A, a redshift of the $\pi -\pi^{*}$ transition is observed, which shifts from 261.83 nm at 1 h of reduction to 276.23 nm at 120 h of reduction (Supplementary Table S4). This redshift is substantially greater than that observed in GO (i.e., 233.3 nm at 24 h of drying time), demonstrating that the reduction process has taken place as well as the structure and characteristics of graphene are gradually being restored.

Figure 1B demonstrates that rGO exhibits a notable rise in absorption in the visible region, with up to a 30% increase at 400 nm and an 11% increase at 700 nm as the reduction time increases. This is in line with what was previously reported in (Kumar et al., 2014) and corroborated in our earlier work (Tene et al., 2023a). Additionally, the improvement in absorption of rGO in the visible region can also be attributed, at least in part, to the presence of OFGs due to:

• Bandgap engineering: GO possesses a relatively large bandgap due to the presence of OFGs disrupting the pristine sp^2^ carbon network. These OFGs introduce energy levels within the bandgap, leading to limited electronic transitions in the visible spectrum. When the material is reduced, these OFGs are removed or reduced, effectively reducing the bandgap. This reduction in bandgap allows the material to absorb a wider range of wavelengths, including those in the visible region.
• The $\pi -\pi^{*}$ transitions: The OFGs in GO introduce additional energy levels that could interfere with or diminish the strength of $\pi -\pi^{*}$ transitions, which are responsible for electronic absorption. As these groups are reduced, the restoration of the π− conjugated system and the removal of energy levels facilitate stronger and more efficient $\pi -\pi^{*}$ transitions, leading to improved visible light absorption.
• Localized states and defects: The OFGs contribute to localized states within the bandgap of GO. These states can act as traps for electrons and hinder the efficient absorption of visible light. During reduction, these localized states are diminished, leading to improved electronic mobility and more efficient light absorption.
• Electronic structure modification: OFGs alter the electronic structure of GO by introducing electron-rich and electron-poor regions. This can lead to uneven charge distribution and hinder efficient absorption. The removal or reduction of these groups in rGO results in a more uniform electronic structure that is conducive to enhanced absorption.
• Influence on exciton dynamics: OFGs can influence the dynamics of excitons (electron-hole pairs) generated upon light absorption. Reduction can alter these dynamics by reducing the presence of quenching sites, leading to improved exciton lifetimes and consequently enhanced absorption.

With this in mind, rGO is a promising material with prospective applications because of its increased absorption in the visible range, such as:

• rGO can efficiently absorb visible light and convert it into electrical energy, which makes it suitable for use in photovoltaics and solar cells.
• rGO can be used as a photocatalyst for visible-light-driven chemical reactions, such as water splitting or CO_2_ reduction, which is important for sustainable energy generation and environmental remediation.
• rGO has potential applications in optoelectronics devices, such as light-emitting diodes (LEDs), where efficient conversion of electrical energy to light is crucial.

Continuing our analysis, we focus on the position of the $\pi -\pi^{*}$ transition. Up to the 48 h of reduction, a consistent linear trend is observed in the position of the $\pi -\pi^{*}$ transition (Figure 1C). However, at this point, the trend starts to level off. This is because the position cannot keep increasing indefinitely; it has inherent limits. To provide context, consider that exfoliated graphene dispersions typically exhibit a maximum position of the $\pi -\pi^{*}$ transition of around 280 nm (Gomez et al., 2021). This comparison highlights that while the linear trend of the reduction process flattens after 48 h, the resulting transition position remains consistent with expectations for similar materials, say, at 120 h of reduction, a maximum position around 276 nm is observed.

Additionally, as the reduction process unfolds over time, a noteworthy trend emerges in the full width at half maximum (FWHM) of the $\pi -\pi^{*}$ transition evident within the rGO samples. This trend is reflected in the progressive increase in the FWHM values, indicative of the widening of the primary absorption peak. These changes are reported in Supplementary Table S4. As well, this increase in the FWHM of the main absorption peak with extended reduction time is a phenomenon that can be attributed to a convergence of influential factors. Among these factors, the enduring presence of functional groups, structural anomalies at the edges and within the plane of the material, and localized domains characterized by a blend of sp^2^-sp^3^ hybridizations all play a contributory role. This interpretation draws reinforcement from the comparative context provided by the evident narrower FWHM observed in the primary absorption peak of exfoliated graphene (Gomez et al., 2021).

By examining the normalized absorbance as a function of FWHM vs. wavelength, Figure 1D illustrates this fact. It is important to note that the spectral weight of the FWHM in rGO is significantly larger than in GO (Supplementary Table S1), indicating that rGO may offer better optical properties with potential for spectroscopic applications.

**FIGURE 2:**
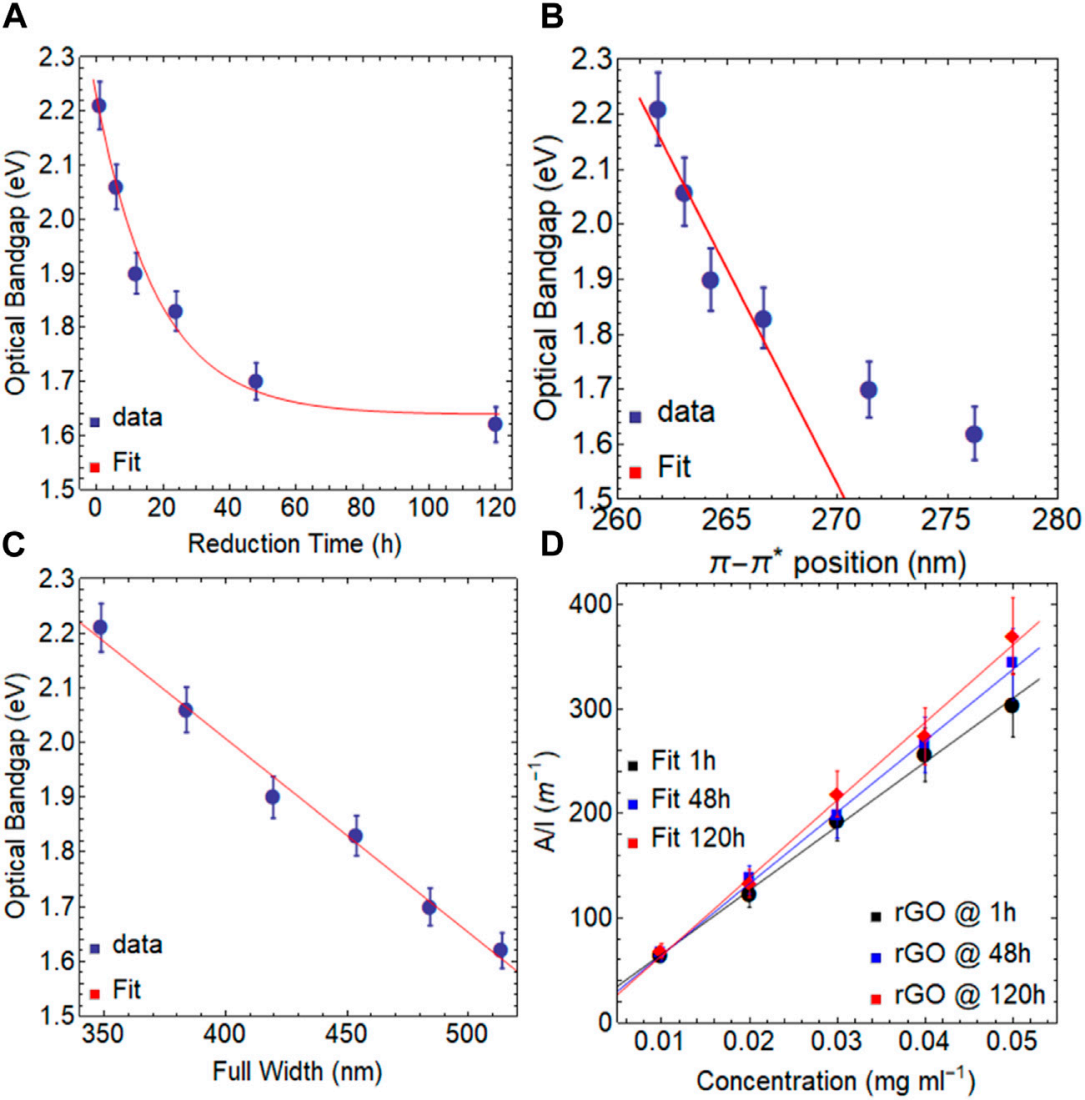
Optical bandgap of rGO at 80 ℃ as a function of **(A)** reduction time, **(B)** position of $\pi -\pi^{*}$ transition, and **(C)** FWHM. **(D)** Optical absorbance ( $\lambda_{660}$ nm) as a function of concentration for different reduction times.

We now discuss the optical bandgap and absorption coefficient of rGO (Tables 2 and 3). Figure 2A depicts the optical bandgap falling from 2.21 eV after 1 h of reduction to 1.62 eV at 120 h of reduction, which can be explained by a decreasing exponential function ( $y\left(t\right)=0.59\;\mathrm{Exp}\left[-5.44\times 10^{-2}\;t\right]+1.64$ ; $R^{2}=0.998$ ). The reduction at 80 °C via AA results in a 0.59 eV overall drop in the optical bandgap.

**TABLE 2:** Estimated optical bandgaps of rGO at 80 ℃ for different reduction times.

Reduction time (h)	Optical bandgap (eV)	*R* ^2^
1	2.21	0.999
6	2.06	0.999
12	1.90	0.999
24	1.83	0.999
48	1.70	0.999
120	1.62	0.999

**TABLE 3:** Estimated optical absorption coefficient of rGO at 80 ℃ for different reduction times.

Material	Absorption coefficient (ml mg^−1^ m^−1^)	*R* ^2^
rGO @ 1 h/80 ℃	6112.98	0.996
rGO @ 48 h/80 ℃	6830.27	0.998
rGO @ 120 h/80 ℃	7425.71	0.994

With a difference of 0.38 eV during the first 24 h of reduction, the optical bandgap decreases most noticeably. After this point, the optical bandgap is still marginally decreased by the reducing agent, but at a much slower rate of $-5.44\times 10^{-2}$ s^−1^, leading to a minimum optical bandgap of 1.64 eV. These findings are significant as they demonstrate that the reduction process can be adjusted to achieve the desired optical bandgap for various applications. Moreover, these results have important implications for scaling up the production of rGO.

Figure 2B displays a quasi-linear relationship between the optical bandgap and the position of the $\pi -\pi^{*}$ transition, which can be written as $y\left(t\right)=-7.81\times 10^{-2}\;t+22.61$ ( $R^{2}=0.901$ ) by data fitting (Table 2). The plot shows an inflection point at 48 h of reduction, which denotes that the optical bandgap and the location of the $\pi -\pi^{*}$ transition have both achieved a saturation point and the reduction has almost completely taken place. Additionally, the slope is $-7.81\times 10^{-2}$ eV nm^−1^, which shows that the optical bandgap is slowly being decreased for each nm of the redshift of the $\pi -\pi^{*}$ transition.

The relationship between the optical bandgap and the FWHM is illustrated in Figure 2C, and it follows a linear pattern represented by the equation: $y\left(t\right)=-3.54\times 10^{-3}\;t+3.42$ ( $R^{2}=0.990$ ). Despite the small slope of $-3.54\times 10^{-3}$ eV nm^−1^, the reduction time significantly influences the FWHM. For example, rGO reduced for 1 h exhibits an optical bandgap of 2.21 eV and an FWHM of 349 nm, while rGO reduced for 120 h displays an optical bandgap of 1.62 eV and an FWHM of 514 nm (Supplementary Table S4). These results emphasize the importance of precisely controlling the reduction time to obtain rGO with desired characteristics.

In Figure 2D, the ratio of A/l (absorbance/path length) displays a linear increase with the rising concentration of rGO, regardless of the reduction time. By analyzing the data, absorption coefficients of 6113.0 ml mg^−1^ m^−1^ at 1 h, 6830.3 ml mg^−1^ m^−1^ at 48 h, and 7425.7 ml mg^−1^ m^−1^ at 120 h were estimated (Table 3). These values are approximately three times higher than those reported for exfoliated graphene dispersions, indicating the superior light absorption capability of rGO. Moreover, when compared to GO, the absorption coefficients of rGO exhibit an increase of approximately 25%.

The notable enhancement in the absorption coefficient of rGO could be attributed to several key factors:

• Conjugated π-electron system: The reduction process of GO involves the restoration of the sp^2^ carbon network, leading to the formation of a highly conjugated π− electron system. This extensive conjugation results in increased delocalization of electrons, facilitating more efficient absorption of light across a wider range of wavelengths.
• Reduced bandgap: GO possesses a relatively wide bandgap due to the presence of OFGs. The reduction of GO into rGO eliminates or reduces these functional groups, effectively narrowing the bandgap. A smaller bandgap enables rGO to absorb a broader spectrum of light, including visible and near-infrared wavelengths.
• Defect-induced absorption: The presence of defects, edges, and structural irregularities in rGO contributes to the creation of localized states within the electronic band structure. These defects can facilitate additional absorption channels, resulting in increased light absorption.
• Interband transitions: The broader π− electron energy levels and the reduced bandgap in rGO create additional interband transitions, allowing absorption of a wider spectrum of light energy.
• Thickness and surface area: The reduction process often leads to the formation of thinner and smaller rGO sheets compared to GO. This reduction in thickness and increased surface area can enhance light-matter interactions and promote higher light absorption.

### 3.3 The case of rGO at 50 ℃

**FIGURE 3:**
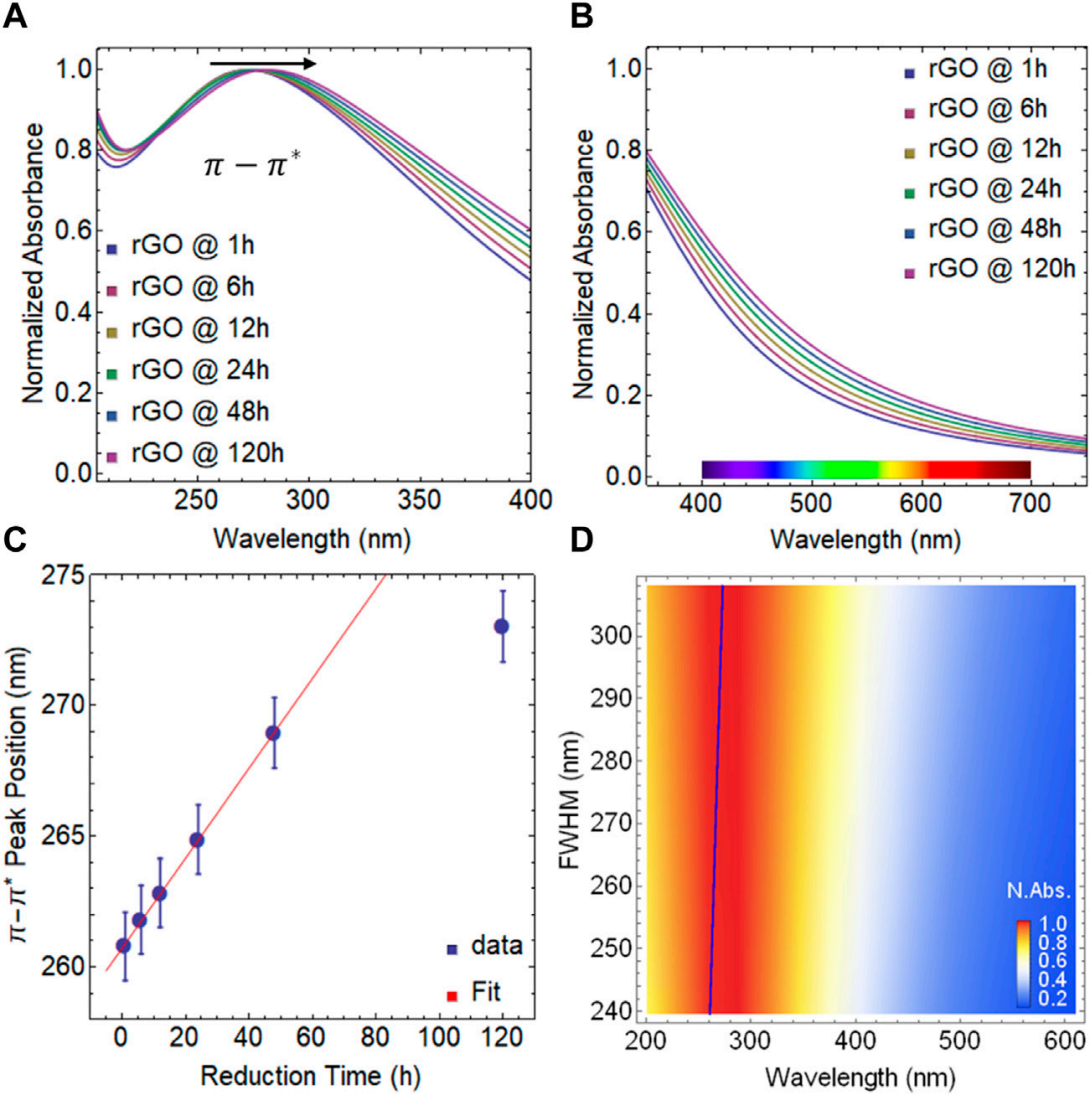
Absorbance spectra of rGO at 50 ℃ : **(A)** from 200–400 nm and **(B)** from 350–750 nm. **(C)** Position of $\pi -\pi^{*}$ transition as a function of reduction time. **(D)** Color plot: Normalized absorbance as a function of the FWHM and wavelength.

In this section, we analyze rGO reduced at 50 °C. The absorbance spectra of rGO for various reduction times are shown in Supplementary Figure S8. While the spectra in the visible region do not display significant features (Supplementary Figure S9), the broad structure of the $\pi -\pi^{*}$ peak remains prominent. Figure 3A indicates a slight redshift from 261 nm at 1 h of reduction to 273 nm at 120 h of reduction (Supplementary Table S5). This smaller shift of the $\pi -\pi^{*}$ transition can be attributed to the fact that the reduction temperature critically influences the kinetics of the reduction process. The reduction of GO at 50°C is slower compared to the reduction at 80°C, resulting in a slighter shift of the $\pi -\pi^{*}$ transition towards longer wavelengths.

In Figure 3B, we observe an increase in light absorption in the visible region for rGO reduced at 50°C. However, the enhancement is notably smaller compared to GO or rGO reduced at 80°C. Specifically, the improvement at 400 nm is approximately 20%, and at 700 nm, it is around 10%. Nevertheless, these findings highlight the versatility of rGO in modifying its interaction with light by adjusting technical parameters, such as the oxidation-reduction processes and the choice of reducing agents used.

Figure 3C shows a plateau in the position of the $\pi -\pi^{*}$ peak, even at 50 °C, indicating that the peak position reaches saturation after 48 h of reduction. This behavior is similar to what is observed for rGO reduced at 80°C. Additionally, as mentioned earlier, the FWHM of the absorbance spectrum increases with longer reduction times in rGO reduced at 50°C (Figure 3D). However, the increase is much smaller compared to rGO reduced at 80°C. For instance, at 1 h, the FWHM is 240 nm, while at 120 h, it reaches 308 nm.

**FIGURE 4:**
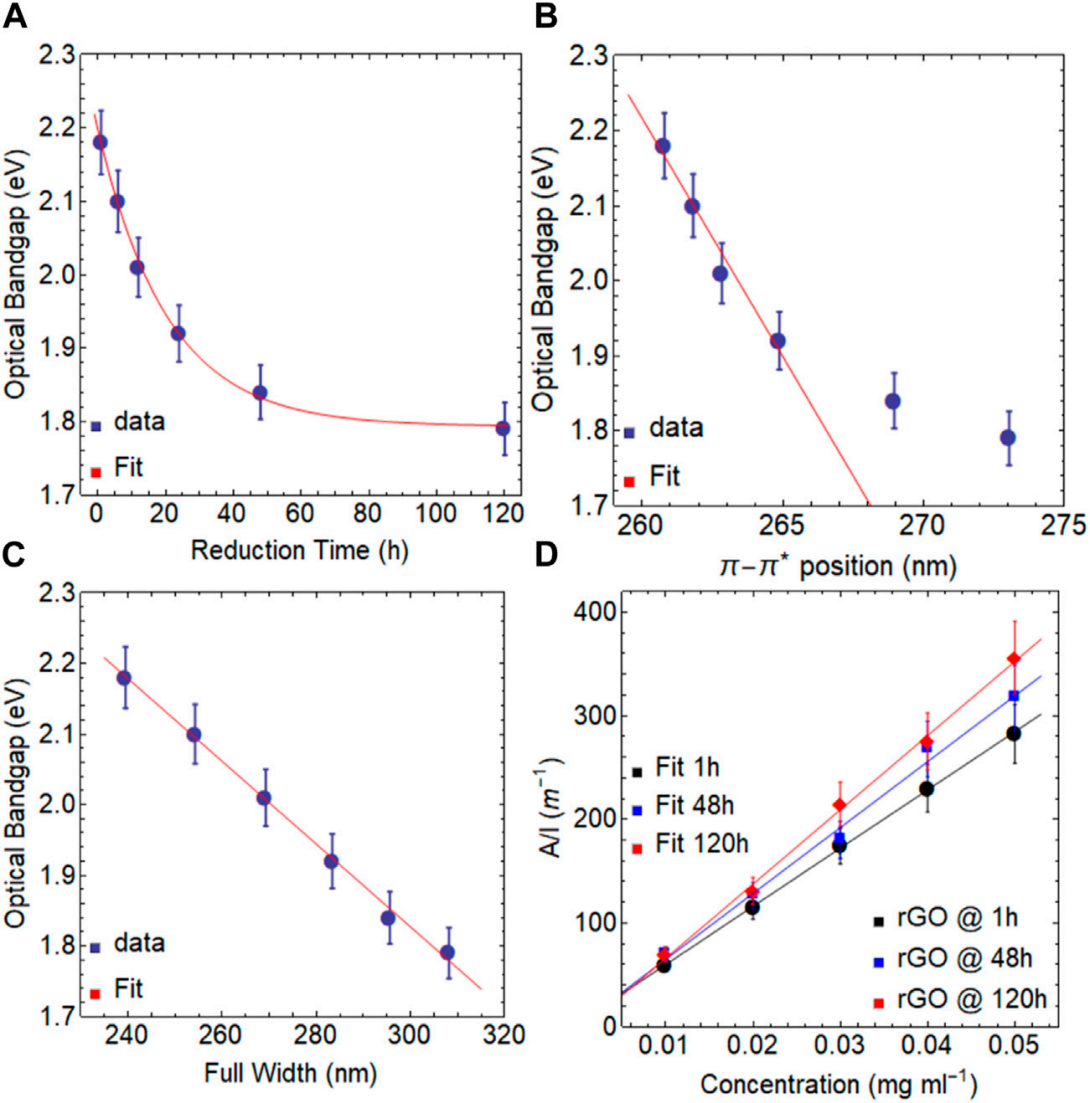
Optical bandgap of rGO at 50 ℃ as a function of **(A)** reduction time, **(B)** position of $\pi -\pi^{*}$ transition, and **(C)** FWHM. **(D)** Optical absorbance ( $\lambda_{660}$ nm) as a function of concentration for different reduction times.

Interestingly enough, when rGO is reduced at 50°C for 1 h (Figure 4A, Supplementary Figure S10A), the resulting optical bandgap point is measured to be 2.18 eV (Table 3), which is slightly lower than the value obtained at 80°C for the same duration (2.21 eV).

Significantly, after 120 h of reduction, the optical bandgap reaches 1.79 eV (Table 4, Supplementary Figure S10F), which is higher than the value observed in the reduction of GO at 80 °C for the same duration (1.62 eV). Additionally, a linear relationship is evident between the optical bandgap and the position of the $\pi -\pi^{*}$ peak (Figure 4B) and FWHM (Figure 4C). This linear trend is particularly pronounced for rGO reduced at 50°C, suggesting that the reduction process is not yet complete, and longer reduction times or higher temperatures may be required to reach the saturation point, similar to what is observed when the reduction is carried out at 80 °C.

**TABLE 4:** Estimated optical bandgaps of rGO at 50 ℃ for different reduction times.

Reduction time (h)	Optical bandgap (eV)	*R* ^2^
1	2.18	0.999
6	2.10	0.999
12	2.01	0.999
24	1.92	0.999
48	1.84	0.999
120	1.79	0.999

The absorbance coefficient of rGO reduced at 50 °C for various reduction times, is reported in Figure 4D and Table 5. The acquired values are lower than those observed for rGO reduced at 80°C (Table 2) but greater than those reported for dispersions of exfoliated graphene and GO (Supplementary Table S3), demonstrating the critical influence of the reduction temperature on the final optical characteristics of the obtained rGO at 50°C.

**TABLE 5:** Estimated optical absorption coefficient of rGO at 50 ℃ for different reduction times.

Material	Absorption coefficient (ml mg^−1^ m^−1^)	*R* ^2^
rGO @ 1 h/50 ℃	5616.44	0.999
rGO @ 48 h/50 ℃	6356.29	0.992
rGO @ 120 h/50 ℃	7159.74	0.997

### 3.4 Spectroscopic and morphological measurements

**FIGURE 5:**
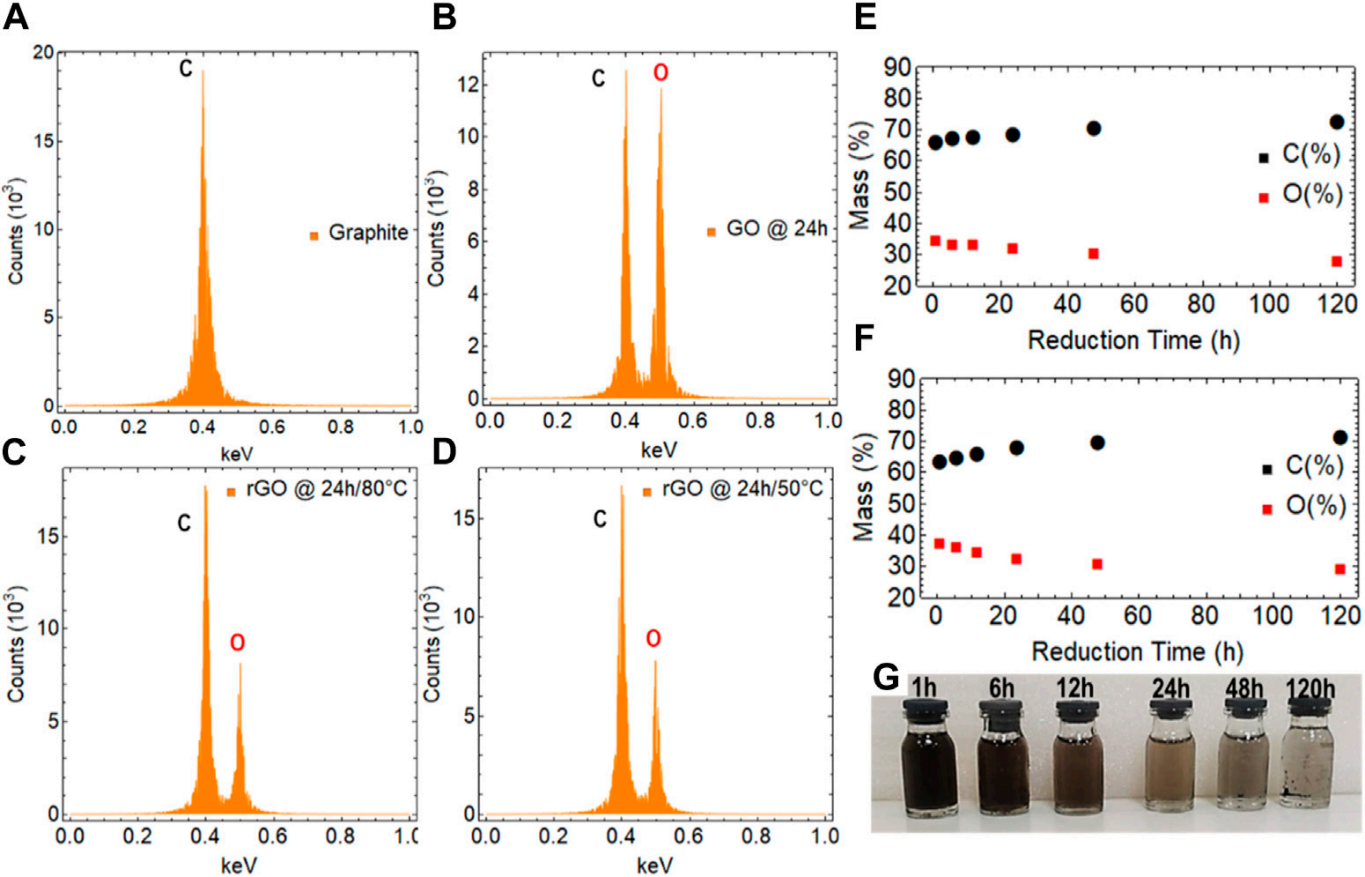
EDS analysis of **(A)** graphite, **(B)** GO dried at 80 ℃ for 24 h, **(C)** rGO at 80 ℃ and 24 h of reduction, and **(D)** rGO at 50 ℃ and 24 h of reduction. Elemental composition of rGO as a function of reduction time **(E)** at 80 ℃ and **(F)** at 50 ℃ . **(G)** Digital images of re-dispersed rGO at 80 ℃ . The rGO dispersions were sonicated in water for 15 min and then left to rest for an additional 15 min before capturing the image.

**FIGURE 6:**
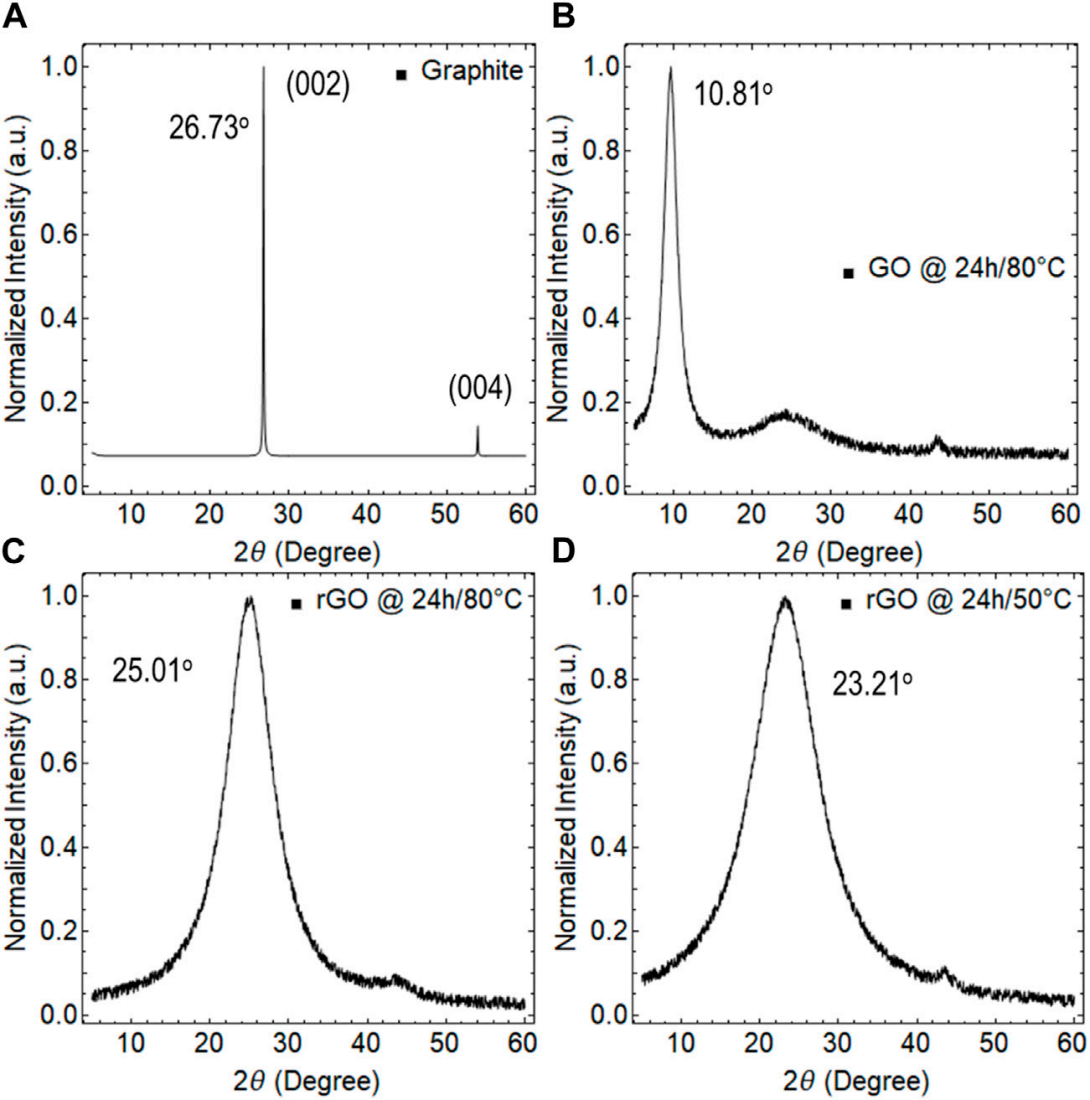
XRD analysis of **(A)** graphite, **(B)** GO dried at 80 ℃ for 24 h, **(C)** rGO at 80 ℃ and 24 h of reduction, and **(D)** rGO at 50 ℃ and 24 h of reduction. The spectra were normalized to the maximum of the prominent peak.

**FIGURE 7:**
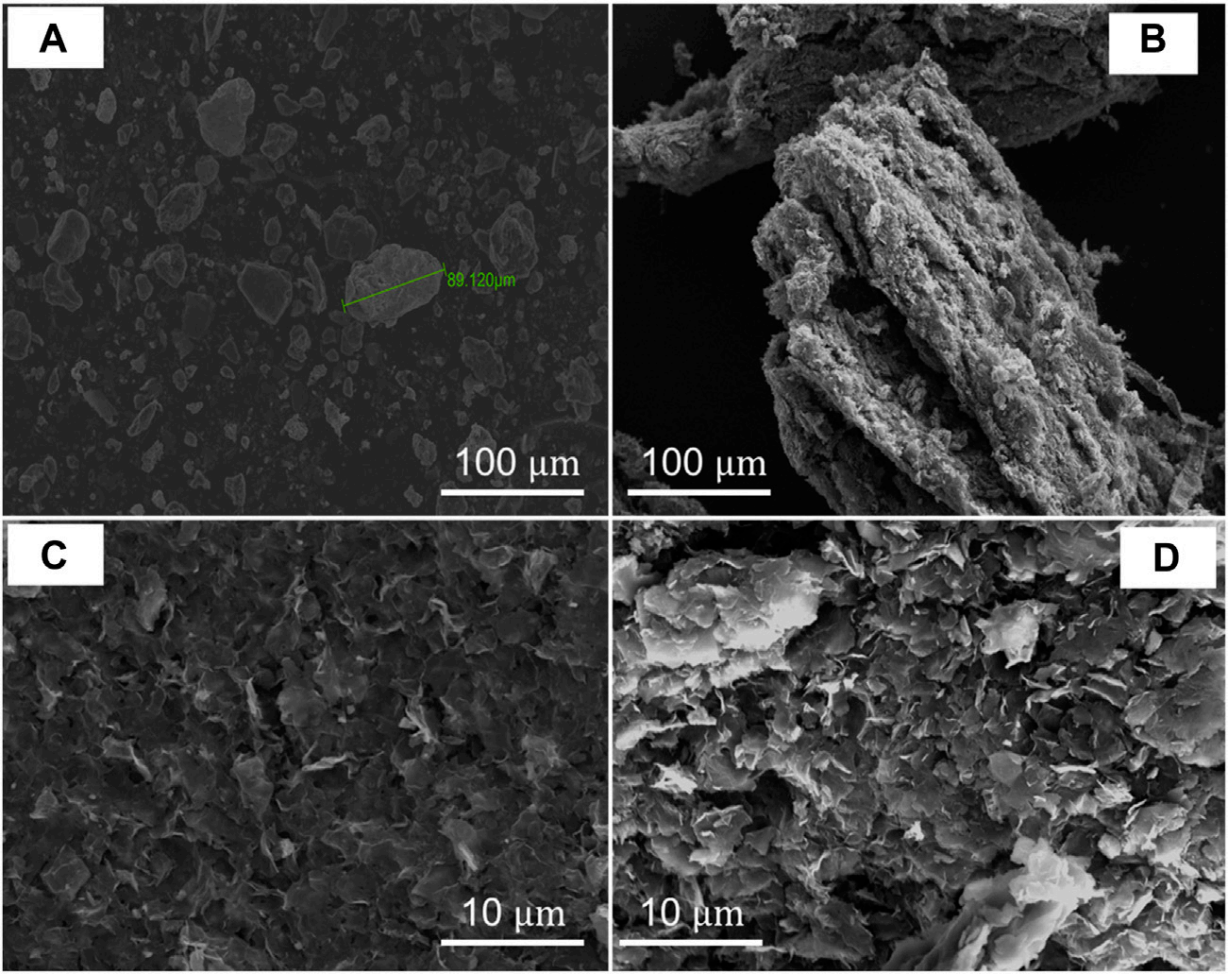
SEM analysis of **(A)** graphite, **(B)** GO dried at 80 ℃ for 24 h, **(C)** rGO at 80 ℃ and 24 h of reduction, and **(D)** rGO at 50 ℃ and 24 h of reduction.

With a focus on samples with 24 h of drying time for GO and 24 h of reduction time for rGO at 50°C and 80°C, we carried out additional studies using EDS (Figure 5), XRD (Figure 6), and SEM (Figure 7) to further complement our findings.

Hence, Figure 5A presents the EDS measurement of graphite, displaying a single peak and a mass percentage of 99.6% (Supplementary Table S6). This peak confirms the presence of only carbon atoms in the sample, aligning with the expected composition of graphite. The high mass percentage of 99.6% indicates that the sample is remarkably pure. On the other hand, Supplementary Figure S11A shows the EDS measurement of GO with a drying time of 0 h, where two peaks are observed for carbon and oxygen with percentage masses of 41.47% and 58.53%, respectively (Supplementary Table S6). These results indicate that the sample is predominantly composed of carbon and oxygen, which is consistent with the expected composition of GO, regardless of the method used for oxidation.

Figure 5B presents the EDS measurement of GO after 24 h of drying time, showing two distinct peaks corresponding to carbon and oxygen, indicating the presence of both elements. The percentage mass of carbon is 51.42%, indicating that it is the major constituent of the sample, while oxygen accounts for 48.58%, signifying a significant amount of oxygen in the material. The decrease in the percentage of oxygen from 58.53% at 0 h of drying time to 48.58% at 24 h confirms that the drying process has effectively removed some of the OFGs from GO. This reduction in oxygen content is essential for the transformation of GO into rGO, as the removal of OFGs plays a key role in restoring sp^2^ hybridization and enhancing the electrical and optical properties of the material.

The EDS measurements on rGO samples reduced at 80°C (Figure 5C) and 50°C (Figure 5D) for 24 h revealed the presence of both carbon and oxygen elements in the obtained materials. In both cases, the carbon peak has a higher percentage mass, with 69.53% at 80°C (Supplementary Table S7) and 69.17% at 50°C (Supplementary Table S8), indicating that carbon is the major constituent of the samples. On the other hand, the oxygen peak in both cases has a lower percentage mass, measuring 30.47% at 80°C and 30.83% at 50°C, which indicates that a considerable amount of oxygen remains in rGO.

These EDS measurements are significant for assessing the effectiveness of the reduction process. Particularly, the results demonstrate that while the reduction process successfully removes a substantial amount of OFGs from GO during the transformation into rGO, a noticeable fraction of oxygen remains in the final material. The presence of oxygen can impact the properties and performance of rGO in various applications, highlighting again the importance of further optimizing the reduction conditions to achieve desired characteristics and properties in eco-friendly-prepared rGO.

Additionally, Figures 5E, F depict the evolution over time of the mass percentage of carbon and oxygen in rGO samples subjected to different reduction times at 80°C (see Supplementary Figure S12) and 50°C (see Supplementary Figure S13). Similarly, the evolution over time of the mass percentage of carbon and oxygen in GO samples subjected to different drying times can be observed in Supplementary Figure S11F. Taking this into consideration, we can observe that when applying the maximum reduction time explored in our current study (120 h) at two distinct temperatures, 80 °C and 50 °C, the resulting rGO samples retain residual oxygen percentages of 26.32% and 27.67% (Supplementary Table S7 and S8), respectively.

The presence of residual oxygen in rGO samples can exert considerable influence on their properties and performance. These oxygen remnants can hinder the electrical conductivity of the material, affecting applications in electronics and sensors. Additionally, the optical properties of rGO may be influenced due to the contribution of residual oxygen, which can impact its potential use in photodetectors and light-emitting devices. Mechanically, the presence of oxygen groups, even at reduced levels, can weaken the overall structural integrity of the material. Moreover, the reactivity of residual oxygen can render rGO more prone to chemical interactions, potentially affecting its stability and suitability for various applications. The surface wettability of rGO might also be influenced by these oxygen groups, altering its interactions with liquids and thus affecting its role in energy storage and catalytic processes.

On the other hand, Figure 5G is particularly significant as it demonstrates the successful recovery of the hydrophobic properties of graphene, confirming the reduction of GO via AA. Specifically, the dispersion of graphene treated at 80°C for 120 h shows clear evidence of precipitates, indicating its low dispersibility in water.

For comparison purposes, Figure 6A shows XRD measurements of graphite, which displays the (002) peak at 26.73° and the (004) peak at 55.2 ° , indicating the high crystalline structure of graphite. In particular, the (002) peak corresponds to the d-spacing between adjacent carbon planes in the graphite structure, which is approximately 3.35 Å .

The XRD measurement of GO dried at 80°C for 24 h (Figure 6B) exhibits a single peak at 10.81°, indicating the presence of an interlayer spacing distance between adjacent GO layers of 7.11 Å. This value is significantly larger than the interlayer spacing distance of graphite (3.35 Å) and can be attributed to the presence of intercalated OFGs. Furthermore, this single peak in the XRD pattern suggests that GO has a disordered structure.

The XRD measurements of rGO reduced at 80°C and 50°C for 24 h are shown in Figures 6C, D, respectively. The relatively narrow peak observed at 25.01° in rGO reduced at 80°C indicates a recovered highly ordered structure with a narrow distribution of interlayer spacing distances between adjacent graphene sheets. On the other hand, the broad peak observed at 23.21° in rGO reduced at 50°C suggests a less ordered structure with a wider distribution of interlayer spacing distances.

The interlayer spacing distances for these peaks are approximately 3.98 Å and 4.24 Å, respectively. These values are significantly smaller than the interlayer spacing distance in GO and can be attributed in fact to the reduction of OFGs and the restoration of sp^2^ hybridization.

The XRD findings are further supported and supplemented by SEM measurements, as depicted in Figure 7. In Figure 7A, the SEM image of graphite showcases a well-defined and regular structure, with a lateral size of less than 100 µm. However, for GO dried for 24 h at 80 °C, a notable transformation in structure is evident in Figure 7B. The image reveals numerous folds within the plane and along the edges, indicating a more complex and irregular surface. Additionally, the surface appears corrugated and lacks the uniformity observed in the graphite sample.

In contrast, the SEM images reveal distinct structural differences among the GO and rGO samples. For instance, rGO reduced at 80°C for 24 h (Figure 7C) exhibits a relatively regular structure with folded edges and a moderate degree of surface roughness. On the other hand, rGO reduced at 50°C (Figure 7D) displays a stacked and disordered structure with numerous folds on the edges. Notably, the SEM image of rGO reduced at 50°C showcases a corrugated surface, which suggests the possible presence of residual OFGs or defects resulting from the reduction process. These observations offer valuable insights into the impact of reduction conditions on the morphology and surface characteristics of rGO, contributing to a comprehensive understanding of the structural properties of the eco-friendly-prepared rGO.

### 3.5 Electrical study

**FIGURE 8:**
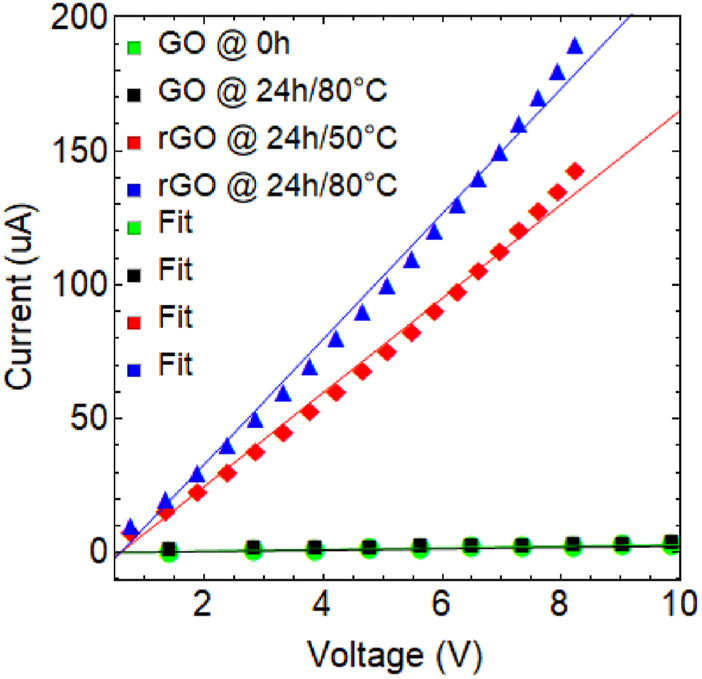
I-V characteristic of non-dried GO (green curve), GO dried at 80 ℃ for 24 h (black curve), rGO at 50 ℃ and 24 h of reduction (red curve), and rGO at 80 ℃ and 24 h of reduction (blue curve).

Lastly, the *I-V* curves (Figure 8) and resistance values (Table 6) obtained for the different samples provide important information about their electrical properties. GO samples dried at 80 °C for 0 h and 24 h (green and black curve, respectively) have the highest resistance ( $3.20\times 10^{6}\;\Omega$ and $3.65\times 10^{6}\;\Omega$ , respectively) among the four samples currently analyzed, indicating poor electrical conductivity which is attributed to the presence of OFGs that act as scattering centers for charge carriers.

**TABLE 6:** Resistance of the as-prepared GO and rGO samples.

Sample	Resistance ( Ω )	*R* ^2^
GO @ 0 h	$3.20\times 10^{6}$	0.990
GO @ 24 h/80 ℃	$3.65\times 10^{6}$	0.995
rGO @ 24 h/50 ℃	$5.70\times 10^{4}$	0.991
rGO @ 24 h/80 ℃	$4.27\times 10^{4}$	0.991

On the other hand, rGO reduced at 50°C (red curve, $5.70\times 10^{4}\;\Omega$ ) and 80°C (blue curve, $4.27\times 10^{4}\;\Omega$ ) for 24 h have significantly lower resistance values (two orders of magnitude less), indicating improved electrical conductivity. These results further confirm the reduction of OFGs and the starting recovery of sp^2^ hybridization, which allows for better charge transport in the graphene-like lattice.

These outcomes can be contextualized for further research and applications in various fields:

i) In the field of electronics, graphene-based materials with high electrical conductivity are highly sought after for the development of efficient and high-performance electronic devices.
ii) In the field of energy storage, rGO with high electrical conductivity can be used as an electrode material in supercapacitors and batteries, where fast charge and discharge rates are essential.
iii) The obtained resistance values can also be used to estimate the sheet resistance of the graphene samples, which is a key parameter for various applications such as transparent conductive films and next-generation sensors.

## 4 Conclusion

In summary, our study has thoroughly examined the optical properties of both GO and rGO, considering various factors related to the oxidation-reduction process such as drying time, reduction time, temperature, and the use of AA as a green reducing agent. Our research has shown that GO has an optical bandgap of approximately 4 eV when dried for 0 h, which gradually decreases to 2.77 eV when dried for 120 h. In contrast, rGO exhibits a reduced bandgap with increased reduction time, with a bandgap of 1.62 eV and 1.79 eV observed for 120 h of reduction time at 80°C and 50°C, respectively. Moreover, the absorbance spectrum of both GO and rGO is mainly characterized by the $\pi -\pi^{*}$ and $n-\pi^{*}$ transitions, which are highly sensitive to the synthesis process parameters mentioned earlier. Interestingly, we found that the FWHM is larger in rGO compared to GO, with a significant spectral weight in the visible region, which is crucial for future applications in sensing and spectroscopy that require high sensitivity to light. Our findings are supported by EDS, XRD, SEM, and electrical measurements, where the resistance of GO was found to be high, indicative of its insulating properties. However, after undergoing the reduction process, the resulting rGO materials showed much lower resistance, indicating that the conductive properties and the sp^2^ carbon network were being recovered.

## Data Availability

The original contributions presented in the study are included in the article/Supplementary Material, further inquiries can be directed to the corresponding author.
