# Supplementary material for: Tunable optical and semiconducting properties of eco-friendly-prepared reduced graphene oxide
Source: Front Chem. 2023 Aug 31;11:1267199. doi: 10.3389/fchem.2023.1267199 (PMC10501135; doi:10.3389/fchem.2023.1267199)
Supplement: Supplementary file 1 [file DataSheet1.docx]

**SUPPLEMENTARY MATERIAL:** **Tunable Optical and Semiconducting Properties of Eco-Friendly-Prepared Reduced Graphene Oxide**

Talia Tene^1^, Yuliana Jiménez-Gaona^1^, Diana Katherine Campoverde-Santo^2^, Yesenia Cevallos^3,4^, Matteo La Pietra^5,6^, Cristian Vacacela Gomez^5,†^, Andrea Scarcello^7,8^, Salvatore Straface^9^, Lorenzo S. Caputi^7,8^, Stefano Bellucci^5^

^1^Department of Chemistry, Universidad Técnica Particular de Loja, Loja 110160, Ecuador

^2^Facultad de Ciencias Pecuarias, Escuela Superior Politécnica de Chimborazo (ESPOCH), 060155, Riobamba, Ecuador.

^3^College of Engineering, Universidad Nacional de Chimborazo, Riobamba 060108, Ecuador

^4^Universidad San Francisco de Quito, Diego de Robles y Vía Interoceánica, Quito 17-1200-841, Ecuador

^5^INFN-Laboratori Nazionali di Frascati, I-00044 Frascati, RM, Italy

^6^Department of Information Engineering, Polytechnic University of Marche, 60131 Ancona, Italy

^7^UNICARIBE Research Center, University of Calabria, I-87036 Rende, CS, Italy

^8^Surface Nanoscience Group, Department of Physics, University of Calabria, Via P. Bucci, Cubo 33C, I-87036 Rende, CS, Italy

^9^Department of Environmental Engineering (DIAm) University of Calabria, Via P. Bucci, Cubo 42B, I-87036 Rende, CS, Italy

† Correspondence to:

C. Vacacela Gomez, Email: vacacela@lnf.infn.it (Corresponding author)

S. Bellucci, Email: stefano.bellucci@lnf.infn.it (Corresponding author)

**
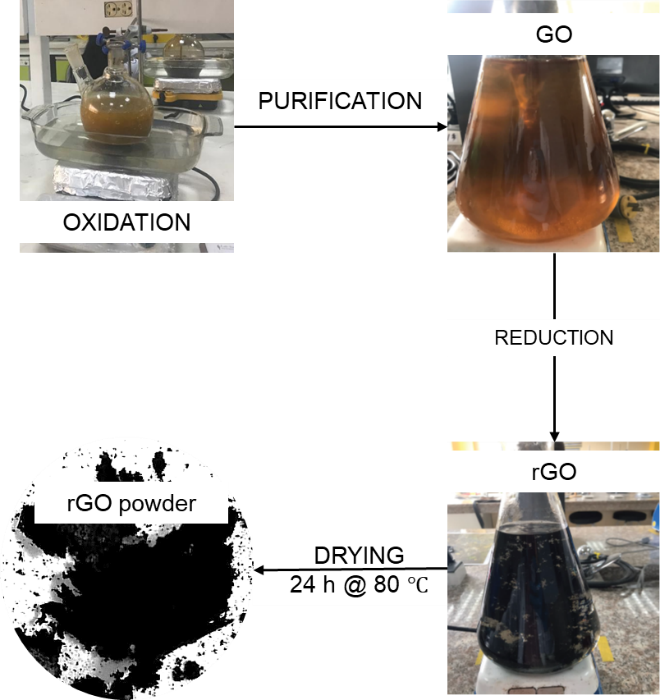
**

**Schematic S1**. Synthesis process for preparing GO and rGO.


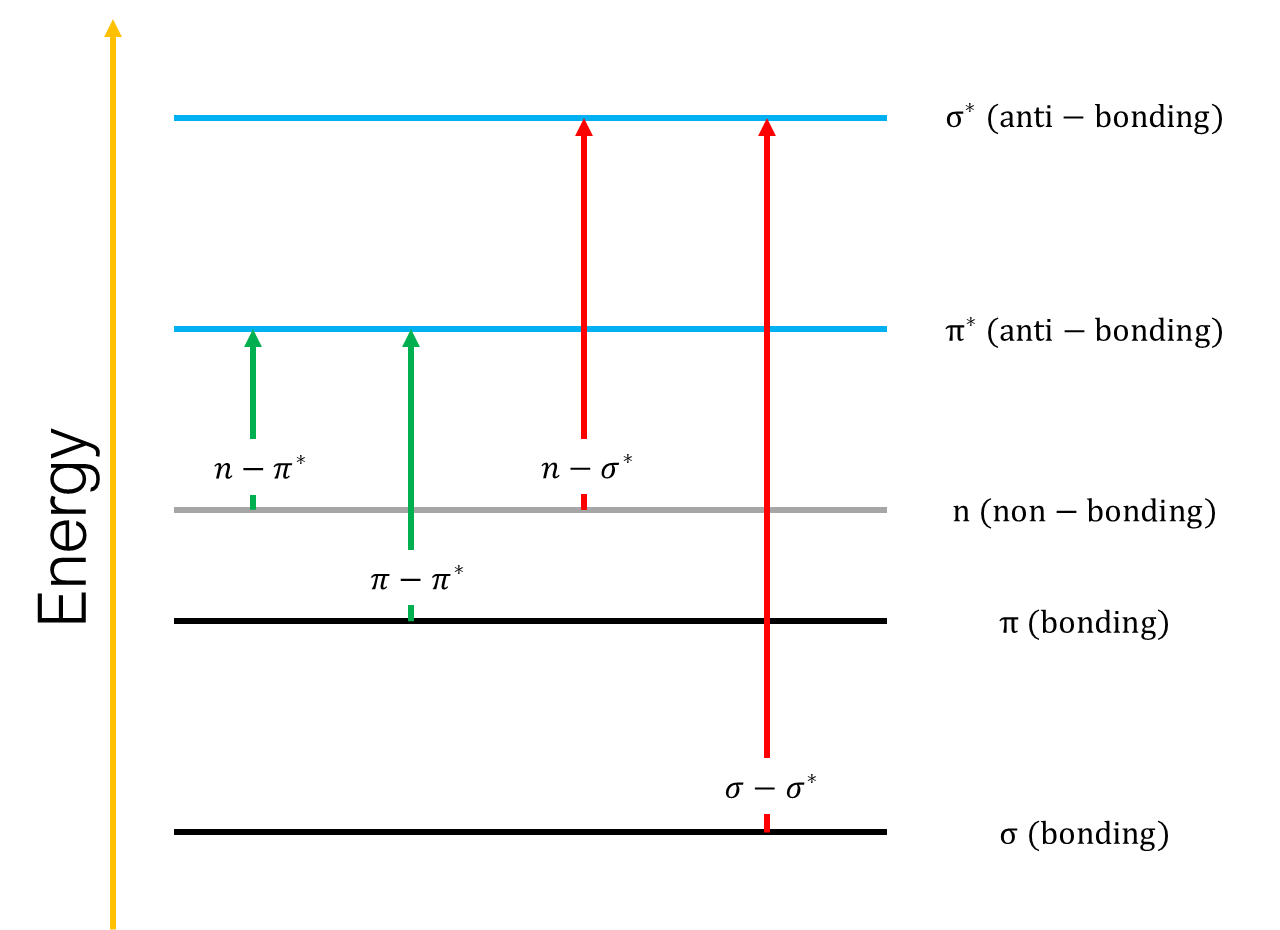


**Schematic S2**. Illustration of UV-visible theory.


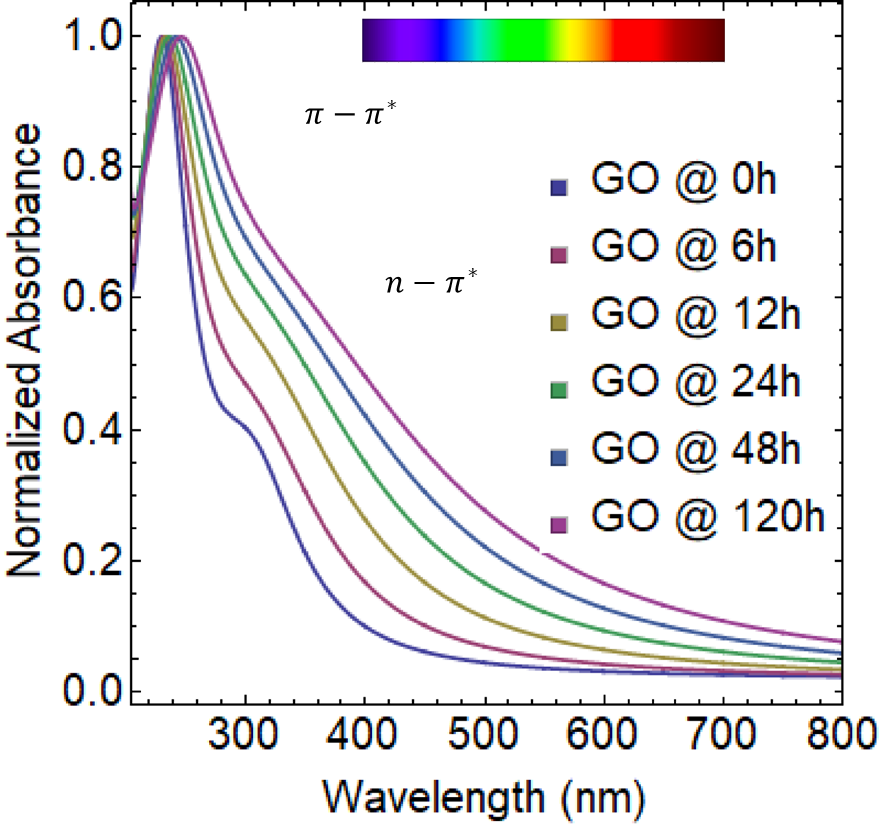


**Figure S1**. Absorbance spectra of GO dried at 80 $℃$ and considering different drying times.


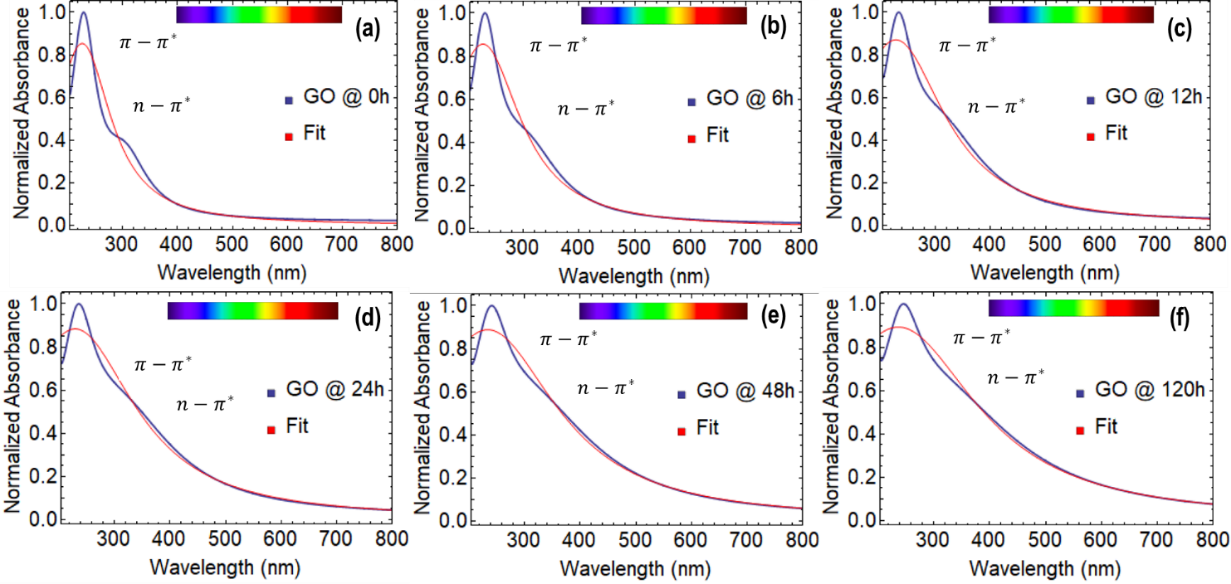


**Figure S2**. Absorbance spectrum of GO at 80 $℃$ considering different drying times: (a) 0h, (b) 6h, (c) 12 h, (d) 24h, (e) 48, and (f) 120h.


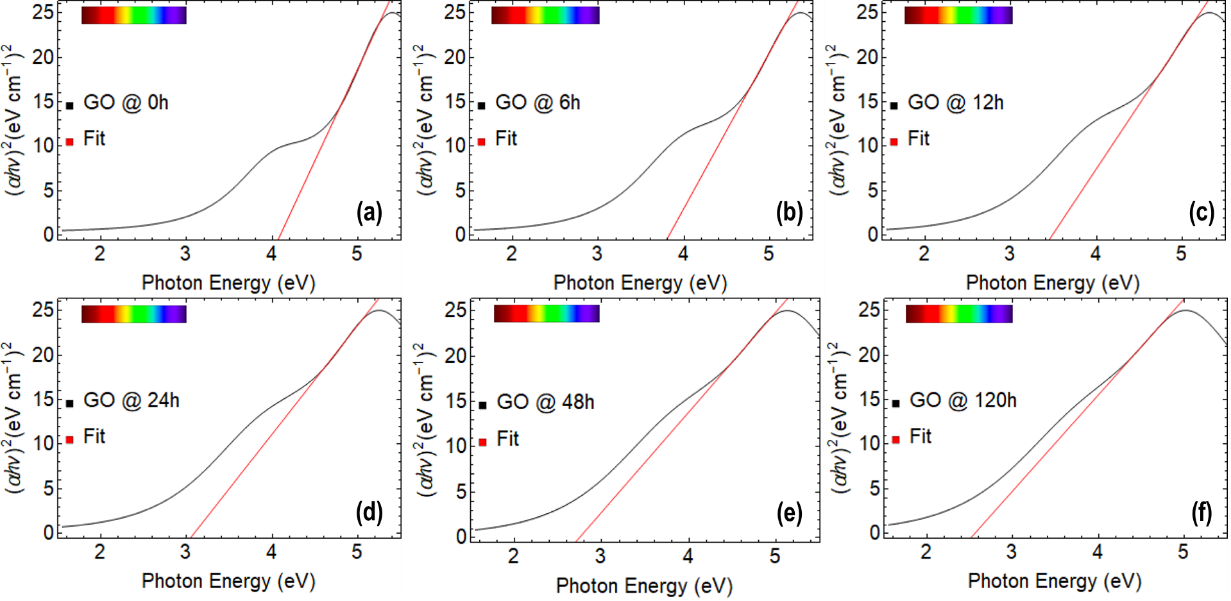


**Figure S3**. Tauc plot of GO at 80 $℃$ considering different drying times: (a) 0h, (b) 6h, (c) 12 h, (d) 24h, (e) 48, and (f) 120h.


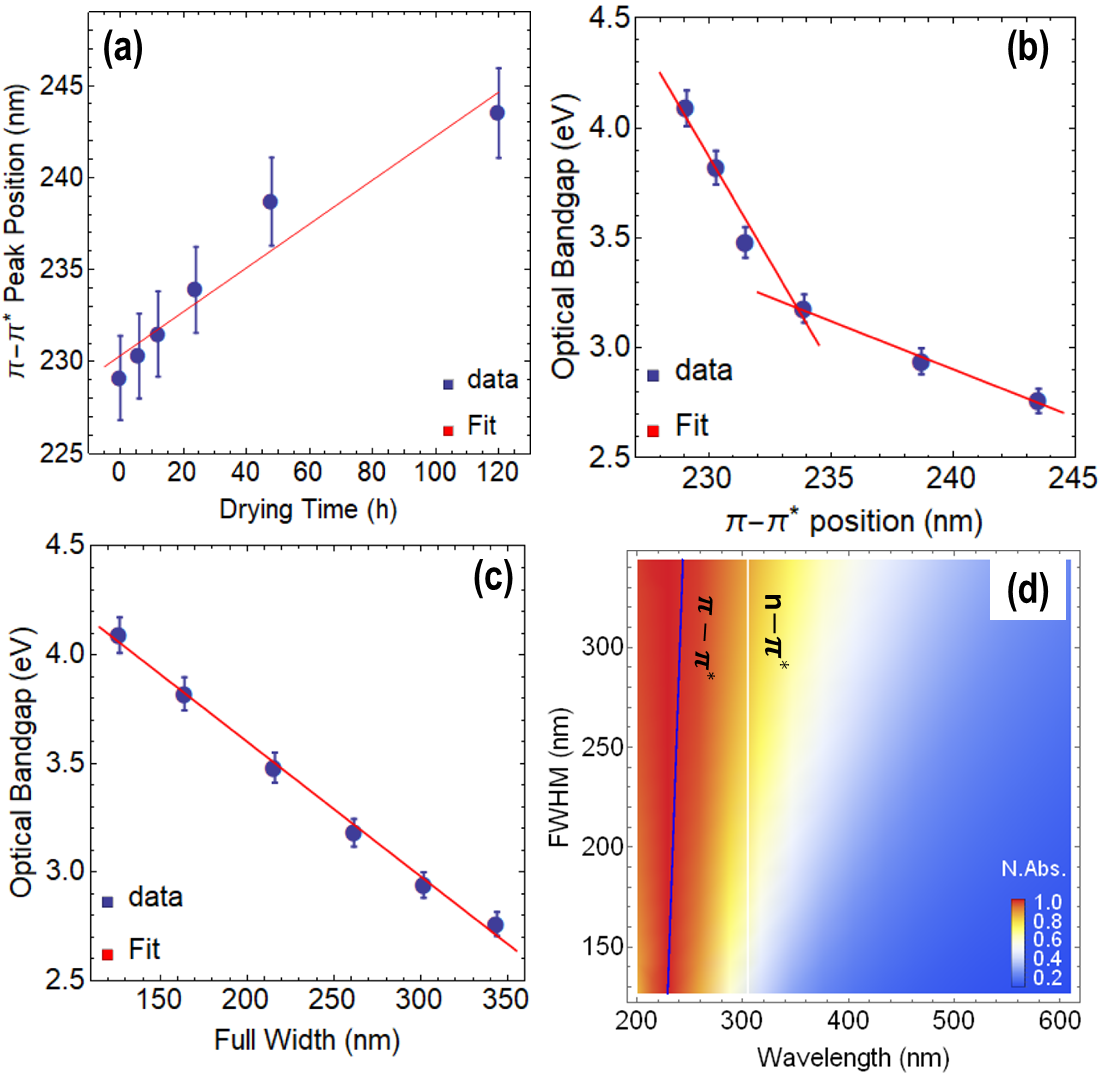


**Figure S4**. GO dried at 80 $℃$. (a) Position of $\pi-\pi^{*}$ transition as a function of drying time. Optical bandgap as a function of: (b) position of $\pi-\pi^{*}$ transition and (c) full-width at half maximum (FWHM). (d) Normalized absorbance as a function of FWHM vs. wavelength


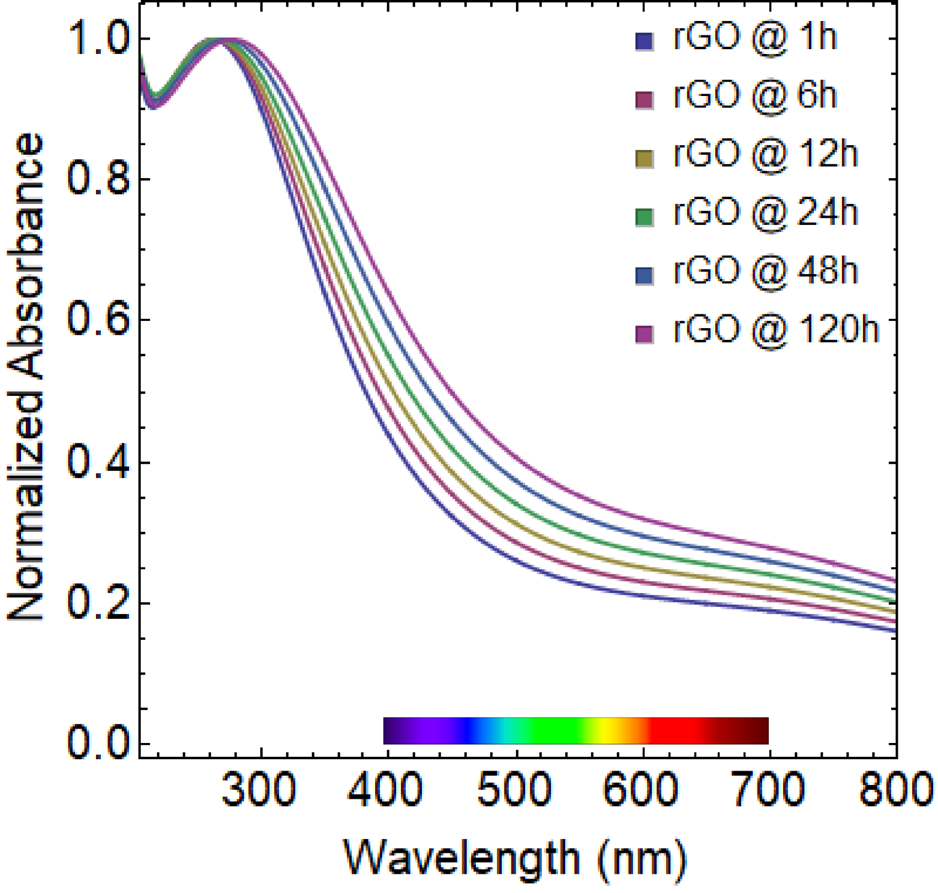


**Figure S5**. Absorbance spectra of rGO reduced at 80 $℃$ and considering different reduction times.


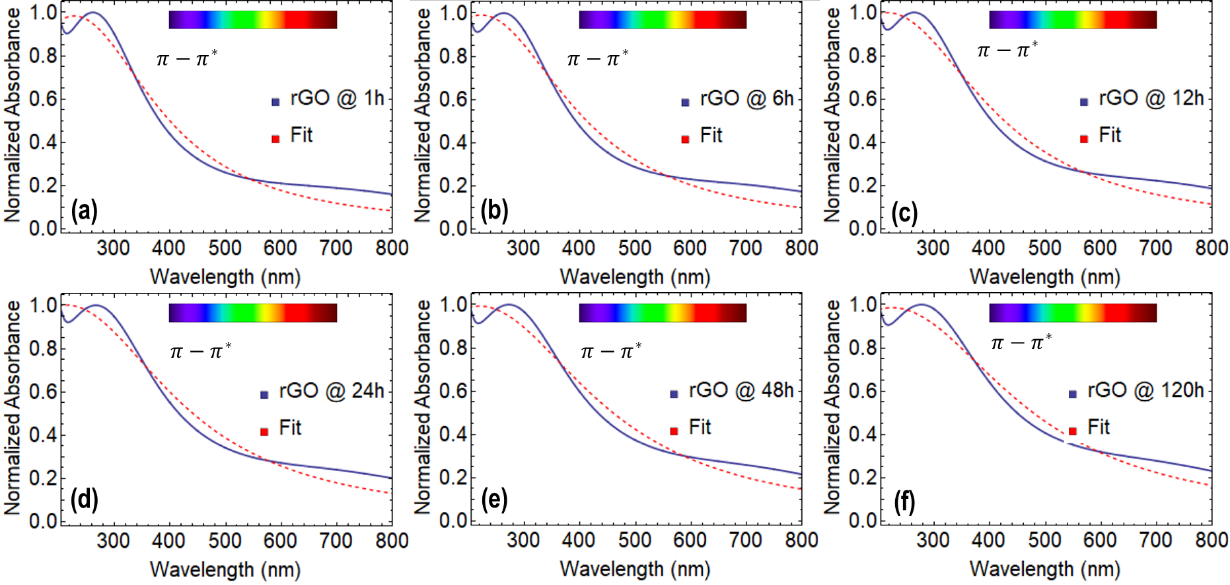


**Figure S6**. Absorbance spectrum of rGO reduced at 80 $℃$ considering different reduction times: (a) 0h, (b) 6h, (c) 12 h, (d) 24h, (e) 48, and (f) 120h.


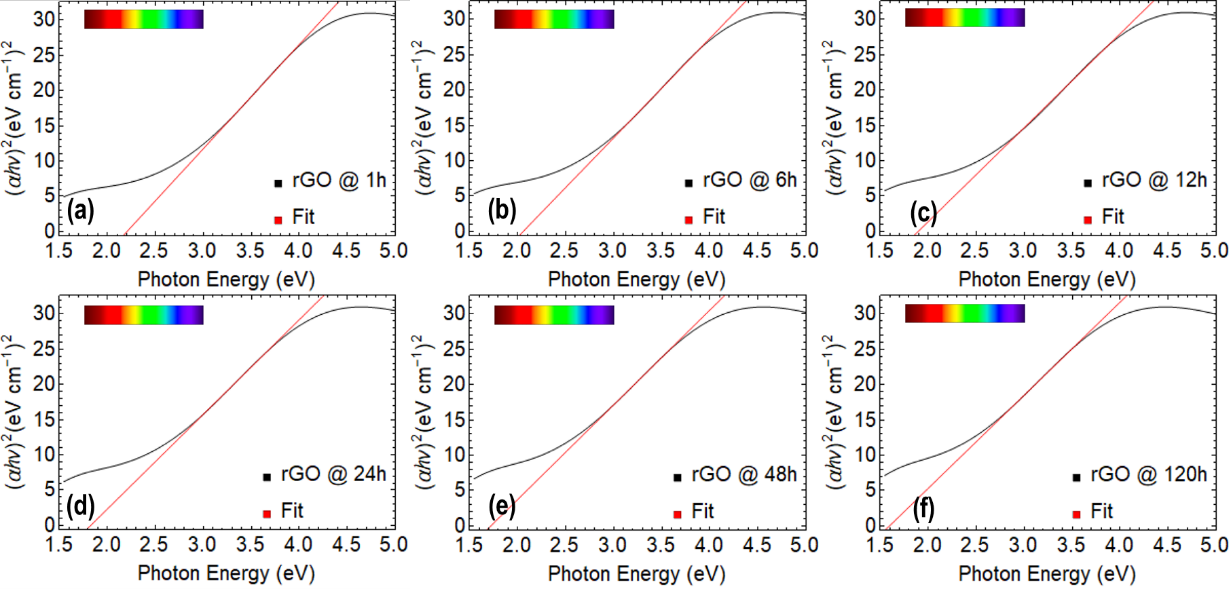


**Figure S7**. Tauc plot of rGO reduced at 80 $℃$ considering different reduction times: (a) 0h, (b) 6h, (c) 12 h, (d) 24h, (e) 48, and (f) 120h.


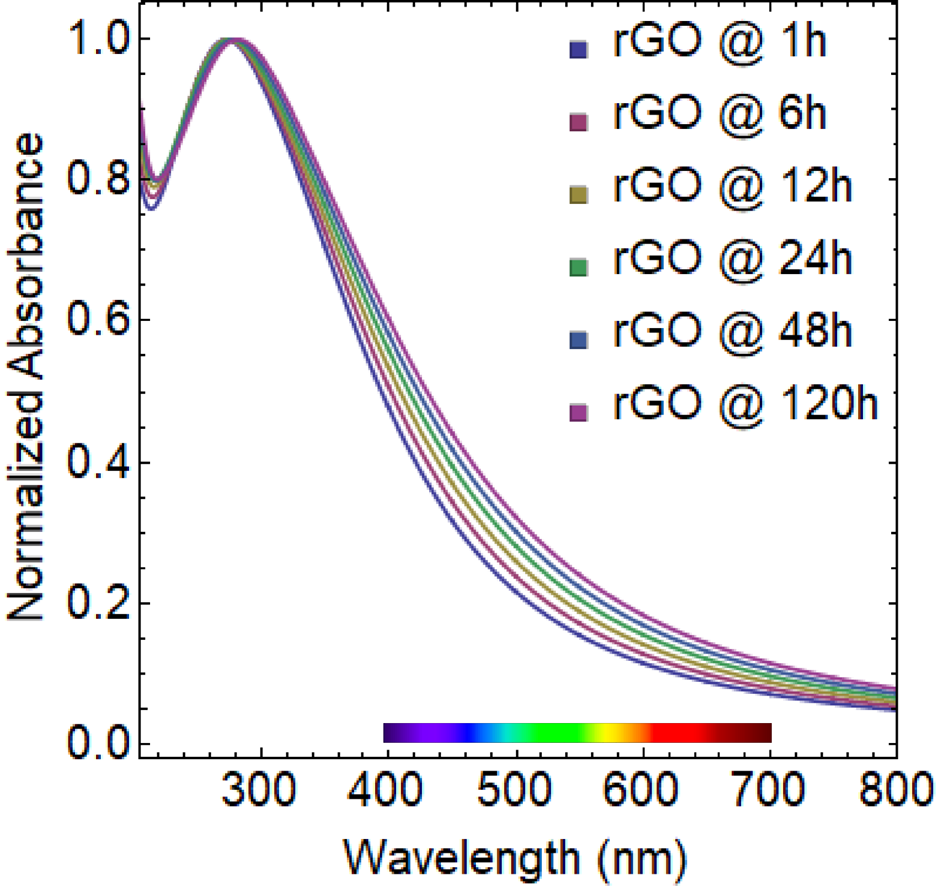


**Figure S8**. Absorbance spectra of rGO reduced at 50 $℃$ and considering different reduction times.


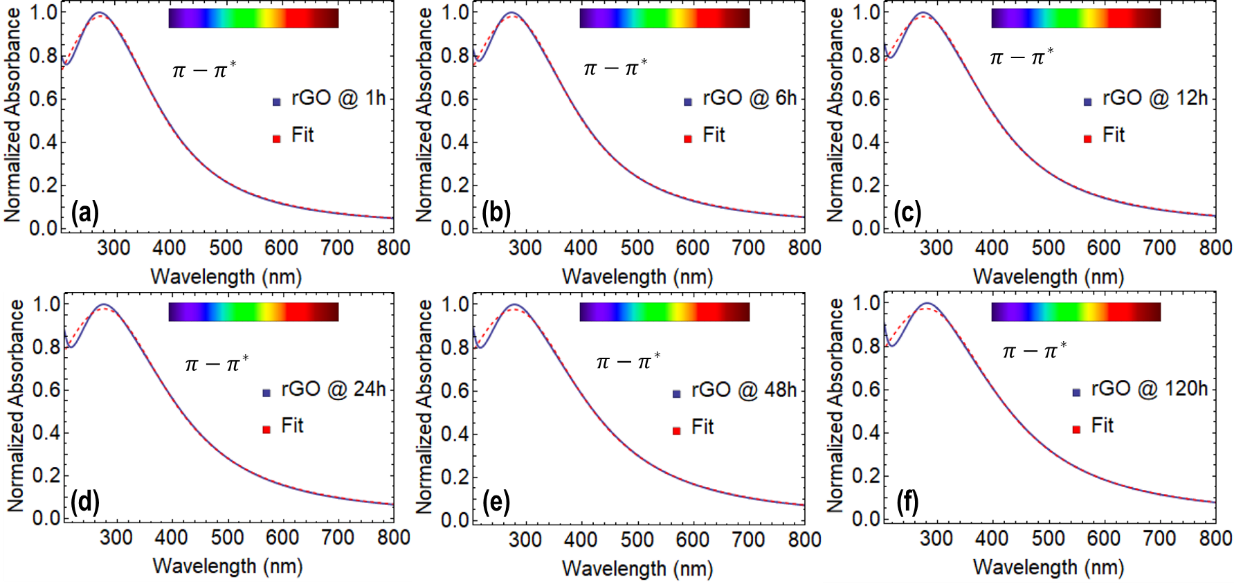


**Figure S9**. Absorbance spectrum of rGO reduced at 50 $℃$ considering different reduction times: (a) 0h, (b) 6h, (c) 12 h, (d) 24h, (e) 48, and (f) 120h.


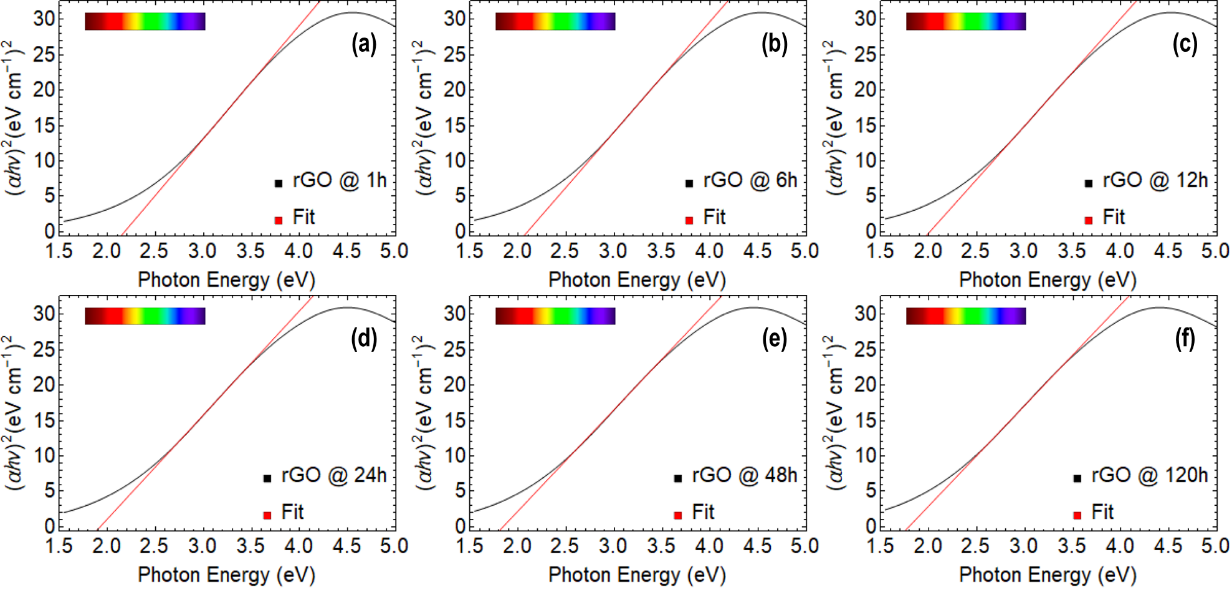


**Figure S10**. Tauc plot of rGO reduced at 50 $℃$ considering different reduction times: (a) 0h, (b) 6h, (c) 12 h, (d) 24h, (e) 48, and (f) 120h


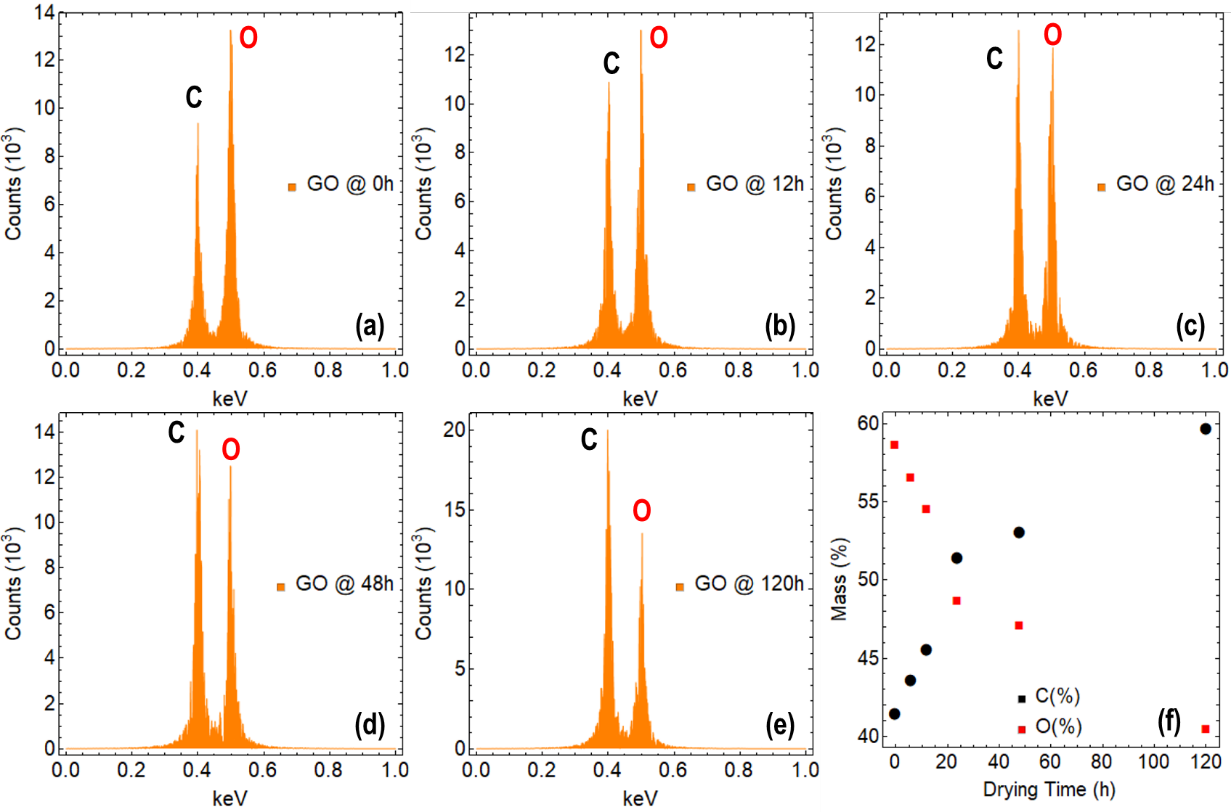


**Figure S11**. EDS measurements of GO dried at 80 $℃$ considering different drying times: (a) 0h, (b) 12 h, (c) 24h, (d) 48, and (e) 120h. (f) Variation of the elemental composition as a function of drying time.


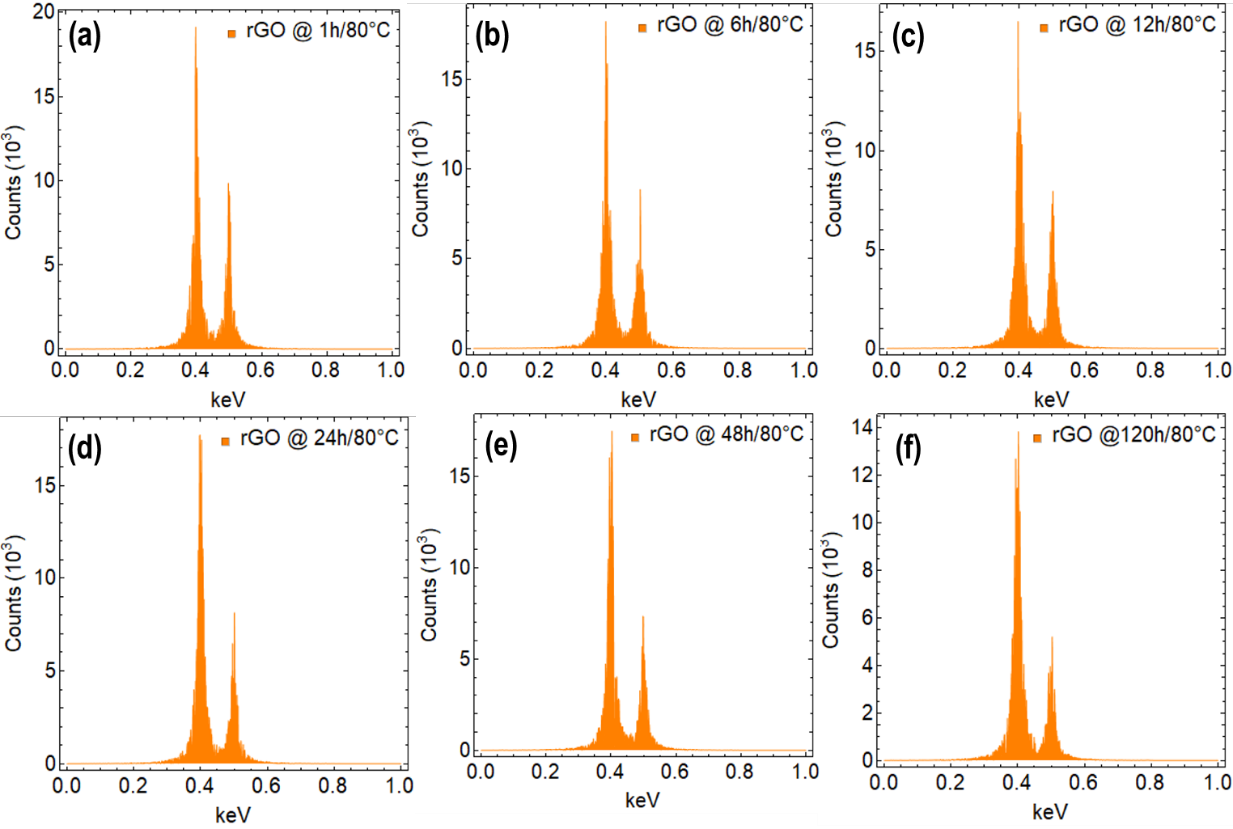


**Figure S12**. EDS measurements of rGO reduced at 80 $℃$ considering different reduction times: (a) 0h, (b) 6h, (c) 12 h, (d) 24h, (e) 48, and (f) 120h.


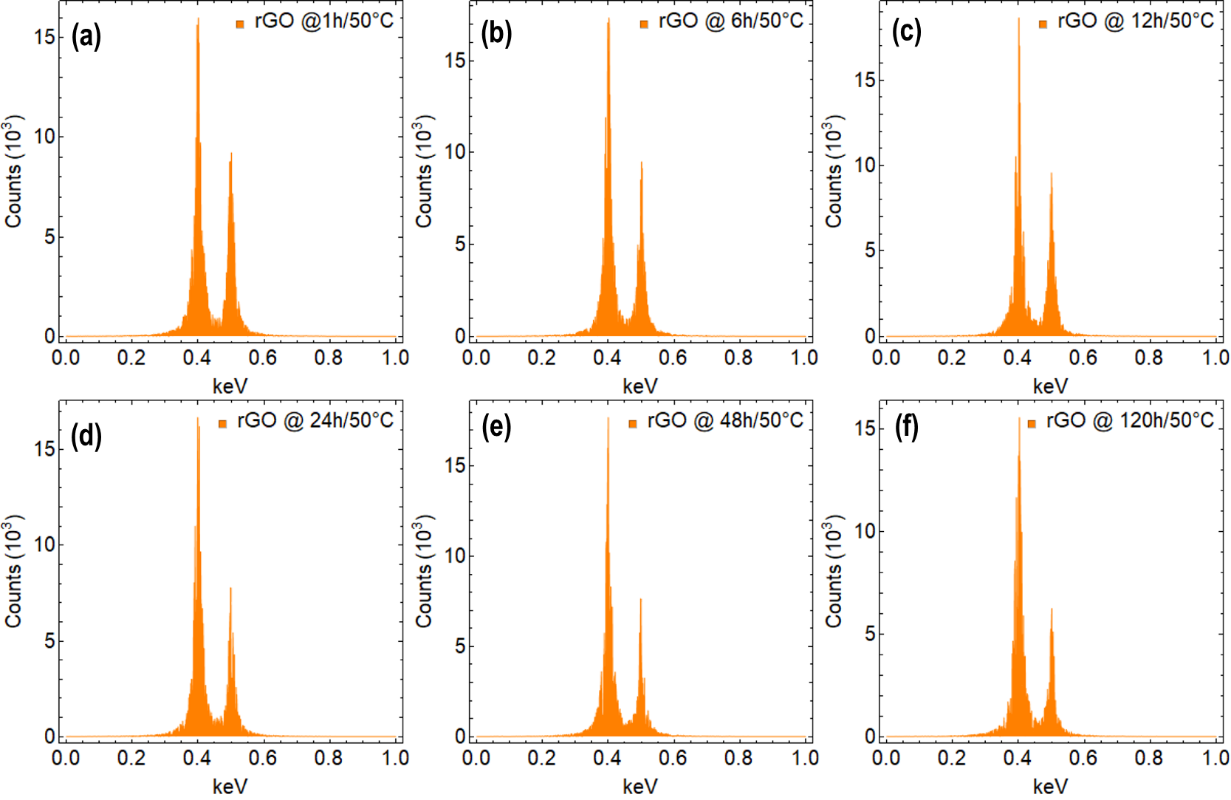


**Figure S13**. EDS measurements of rGO reduced at 50 $℃$ considering different reduction times: (a) 0h, (b) 6h, (c) 12 h, (d) 24h, (e) 48, and (f) 120h.

**Table S1.** Peak Position of $\pi-\pi^{*}$ and $n-\pi^{*}$transitions in GO dried at 80 $℃$ and related full width at half maximums (FWHM), considering different drying times.

| **Drying Time (h)** | $\boldsymbol{\pi-}\boldsymbol{\pi}^{\boldsymbol{*}}$ **Transition (nm)** | $\boldsymbol{n-}\boldsymbol{\pi}^{\boldsymbol{*}}$ **Transition (nm)** | **FWHM (nm)** | **R^2^** |
| --- | --- | --- | --- | --- |
| 0 | 229.63 | 305.15 | 126.57 | 0.982 |
| 6 | 230.59 | 305.15 | 163.92 | 0.987 |
| 12 | 231.51 | 305.16 | 215.92 | 0.992 |
| 24 | 233.23 | 305.17 | 261.59 | 0.994 |
| 48 | 238.23 | 305.19 | 301.72 | 0.995 |
| 120 | 243.81 | 305.27 | 343.90 | 0.996 |

**Table S2.** Estimated optical bandgap values of GO dried at 80 $℃$ as a function of drying time.

| **Drying Time (h)** | **Optical Bandgap (eV)** | **R^2^** |
| --- | --- | --- |
| 0 | 4.09 | 0.996 |
| 6 | 3.82 | 0.996 |
| 12 | 3.48 | 0.998 |
| 24 | 3.18 | 0.998 |
| 48 | 2.94 | 0.998 |
| 120 | 2.76 | 0.999 |

**Table S3.** Optical absorption coefficient of GO dried at 80 $℃$ estimated by a linear fit of the optical absorbance over cell length as a function of concentration under three drying times.

| **Material** | **Absorption coefficient (ml mg^-1^ m^-1^)** | **R^2^** |
| --- | --- | --- |
| GO @ 0 h | 3932.22 | 0.992 |
| GO @ 48 h | 4586.71 | 0.992 |
| GO @ 120 h | 5507.15 | 0.985 |

**Table S4.** Peak Position of the $\pi-\pi^{*}$ transition in rGO reduced at 80 $℃$ and related full width at half maximums (FWHM), considering different reduction times.

| **Drying Time (h)** | $\boldsymbol{\pi-}\boldsymbol{\pi}^{\boldsymbol{*}}$ **Transition (nm)** | **FWHM (nm)** | **R^2^** |
| --- | --- | --- | --- |
| 1 | 261.83 | 348.79 | 0.990 |
| 6 | 263.03 | 383.93 | 0.991 |
| 12 | 264.23 | 419.62 | 0.991 |
| 24 | 266.63 | 453.89 | 0.992 |
| 48 | 271.43 | 484.24 | 0.993 |
| 120 | 276.23 | 513.85 | 0.994 |

**Table S5.** Peak Position of the $\pi-\pi^{*}$ transition in rGO reduced at 50 $℃$ and related full width at half maximums (FWHM), considering different reduction times.

| **Drying Time (h)** | $\boldsymbol{\pi-}\boldsymbol{\pi}^{\boldsymbol{*}}$ **Transition (nm)** | **FWHM (nm)** | **R^2^** |
| --- | --- | --- | --- |
| 1 | 260.79 | 239.49 | 0.999 |
| 6 | 261.81 | 254.26 | 0.999 |
| 12 | 262.83 | 269.26 | 0.999 |
| 24 | 264.87 | 283.37 | 0.999 |
| 48 | 268.95 | 295.61 | 0.999 |
| 120 | 273.03 | 308.14 | 0.999 |

**Table S6.** Elemental composition of graphite and GO dried at 80 $℃$, considering different drying times.

| **Sample** | **C (%)** | **O (%)** |
| --- | --- | --- |
| Graphite | 99.61 | --- |
| GO @ 0 h | 41.47 | 58.53 |
| GO @ 6 h | 43.75 | 56.25 |
| GO @ 12 h | 45.56 | 54.44 |
| GO @ 24 h | 51.42 | 48.58 |
| GO @ 48 h | 53.01 | 46.99 |
| GO @ 120 h | 59.65 | 40.35 |

**Table S7.** Elemental composition of rGO reduced at 80 $℃$, considering different reduction times.

| **Sample** | **C (%)** | **O (%)** |
| --- | --- | --- |
| rGO @ 1 h | 66.99 | 33.01 |
| rGO @ 6 h | 68.28 | 31.72 |
| rGO @ 12 h | 68.52 | 31.48 |
| rGO @ 24 h | 69.53 | 30.47 |
| rGO @ 48 h | 71.44 | 28.56 |
| rGO @ 120 h | 73.68 | 26.32 |

**Table S8.** Elemental composition of rGO reduced at 50 $℃$, considering different reduction times.

| **Sample** | **C (%)** | **O (%)** |
| --- | --- | --- |
| rGO @ 1 h | 64.42 | 35.58 |
| rGO @ 6 h | 65.59 | 34.41 |
| rGO @ 12 h | 67.06 | 32.94 |
| rGO @ 24 h | 69.17 | 30.83 |
| rGO @ 48 h | 70.83 | 29.17 |
| rGO @ 120 h | 72.33 | 27.67 |
